# GAMMA: gap-aware motif mining under incomplete labeling with applications to MHC motifs

**DOI:** 10.1093/bioinformatics/btag014

**Published:** 2026-01-14

**Authors:** Xinyi Tang, Ran Liu

**Affiliations:** Department of Mathematics, Statistics and Insurance, The Hang Seng University of Hong Kong, Shatin, Hong Kong SAR, China; Department of Statistics, The Chinese University of Hong Kong, Shatin, Hong Kong SAR, China; Department of Statistics, Faculty of Arts and Sciences, Beijing Normal University, Zhuhai 519087, China

## Abstract

**Motivation:**

Sequence motif identification is crucial for understanding molecular recognition, particularly in immune responses involving peptide binding to major histocompatibility complex (MHC) Class I molecules for antigen presentation to T cells. Traditionally, MHC Class I binding motifs are assumed to be contiguous and span nine amino acids. However, structural evidence suggests that binding may involve nonadjacent residues, challenging the assumptions of existing methods.

**Results:**

In this study, we propose Gap-Aware Motif Mining Algorithm (GAMMA), a probabilistic framework designed to identify noncontiguous motifs under conditions of incomplete labeling. GAMMA employs Bayesian inference with Markov chain Monte Carlo sampling to jointly estimate motif parameters, binding locations, and the relative spacing between binding positions. Through extensive simulations and real-world applications to MHC Class I peptide datasets, GAMMA outperforms existing motif discovery tools such as GLAM2 in accurately localizing binding residues and identifying the underlying motifs. Notably, our results suggest that the true number of binding residues may be eight, fewer than the commonly assumed nine. In addition, for longer peptides, the model captures increased flexibility in the central region, consistent with structural observations that peptides may bulge in the middle.

**Availability and implementation:**

The raw data and the source codes are available on GitHub (https://github.com/RanLIUaca/GAMMAmotif).

## 1. Introduction

Accurate identification of sequence motifs is central to understanding molecular recognition in biological systems. Motifs, defined as short and conserved patterns within biological sequences, play a crucial role in regulating and mediating interactions at the molecular level (Bailey *et al.* 2015). Their discovery has enabled advances in diverse areas, including transcription factor binding (Inukai *et al.* 2017, Tognon *et al.* 2023), protein–protein interactions (Tompa *et al.* 2014), and antigen recognition (Rapin *et al.* 2008, Tadros *et al.* 2023).

In the context of immune responses, motifs are particularly important in peptide binding to major histocompatibility complex (MHC) molecules. MHCs present short peptide fragments on the cell surface, guiding immune surveillance and recognition (Wu *et al.* 2002, Murphy and Weaver 2016). The specific binding preferences of MHC molecules are often governed by conserved sequence motifs within the peptide, which help determine which antigens are effectively displayed to initiate downstream immune activity (Peters *et al.* 2020).

Most existing MHC–peptide binding prediction algorithms assume that the binding motif corresponds to a contiguous subsequence of fixed length, typically nine amino acids. This assumption is embedded in the design of many scoring schemes and alignment-based models (Sette *et al.* 1989, Nielsen and Lund 2009, Bassani-Sternberg *et al.* 2017, Reynisson *et al.* 2020, Gfeller *et al.* 2023, Kushwaha *et al.* 2024), which search for fixed-length, gapless patterns within peptides.

However, structural data from the Protein Data Bank (PDB) (Berman *et al.* 2000) have shown that MHC molecules can bind to peptides in a more flexible manner, especially when the peptides are longer than nine residues. In such cases, the actual binding positions are often not continuous but scattered across the peptide sequence. Despite this evidence, most computational models still rely on simplified assumptions, such as enforcing fixed-length contiguous motifs or truncating longer peptides, which may fail to capture the full diversity of binding modes.

This discrepancy leads to two important modeling questions:

1. How can prediction models be adapted to account for noncontiguous binding patterns observed in longer peptides?
2. Could the true binding motif be shorter than nine residues but separated by gaps, leading to an apparent preference for nine-residue peptides in experimental datasets?

**Figure 1:**
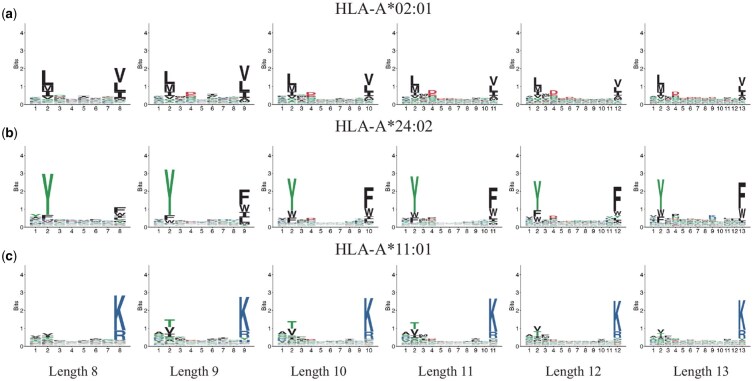
Sequence logos of MHC-bound peptides with different lengths. Subfigures (a) to (c) correspond to three common HLA Class I alleles: (a) HLA-A*02:01, (b) HLA-A*11:01, and (c) HLA-A*24:02. Within each subfigure, peptides are grouped by length (8–13 amino acids). The height of each letter indicates the information content at that position, measured in bits.

**Figure 2:**
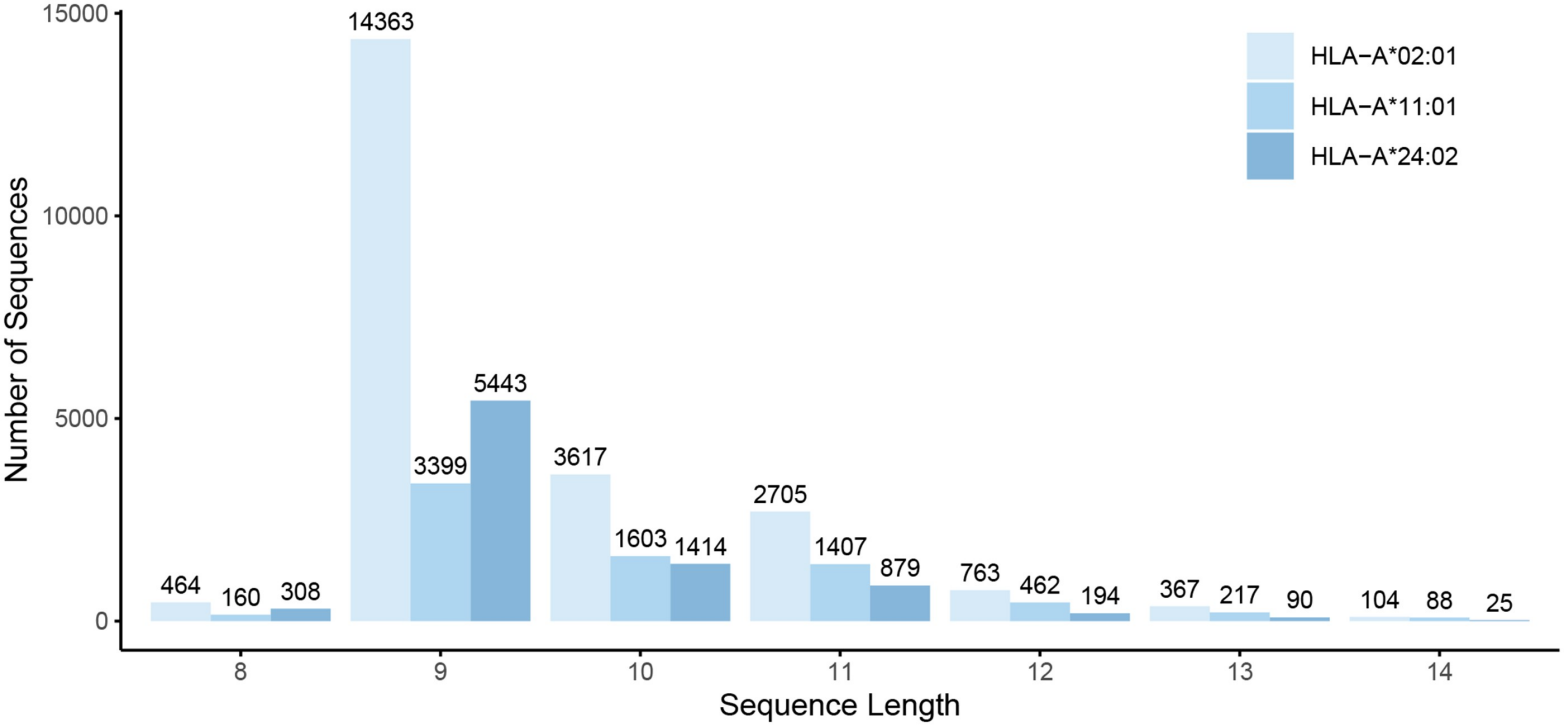
Length distribution of MHC-bound peptides. Histogram showing the distribution of peptide lengths for three representative HLA Class I alleles (HLA-A*02:01, HLA-A*11:01, and HLA-A*24:02). The *y*-axis represents the number of peptide sequences observed for each length.

Figures 1 and 2 present the sequence logos and peptide length distributions, respectively, for MHC-bound peptides derived from the MHC Motif Atlas (Tadros *et al.* 2023). The selected MHC alleles, HLA-A*02:01, HLA-A*11:01, and HLA-A*24:02, are among the most prevalent in the Chinese population (He *et al.* 2018). As shown in the figures, only one or two positions within the peptide sequences exhibit strong conservation for each allele. This observation raises the possibility that the core binding motif may consist of just a few key residues, with flexible or variable-length spacers in between.

This hypothesis aligns with structural and biochemical evidence, which further challenges the notion of a fixed, contiguous motif. Studies have shown that critical binding residues in MHC-peptide interactions are not adjacent in sequence (Adams *et al.* 2016); rather, binding residues can be dispersed and separated by gaps of varying lengths. Such structural flexibility implies that the true binding determinants may be poorly captured by traditional motif models. As a result, models that assume contiguous binding motifs may oversimplify the underlying interaction, potentially limiting both interpretability and generalizability across peptides of varying lengths and different MHC alleles.

To address these limitations, we propose Gap-Aware Motif Mining Algorithm (GAMMA), a probabilistic framework designed to capture flexible, noncontiguous motifs in peptide sequences, particularly under conditions of incomplete labeling. Unlike traditional motif discovery tools for MHC motifs, GAMMA explicitly models the spacing of binding residues using Bayesian inference with Markov chain Monte Carlo (MCMC) sampling. This approach allows for the identification of binding residues that may be separated by variable-length gaps, offering a more realistic representation of MHC–peptide binding preferences. In the following sections, we describe the design of GAMMA in detail, validate its performance against existing methods using synthetic and real-world datasets, and demonstrate its utility in uncovering biologically meaningful patterns across diverse MHC alleles.

## 2. Materials and methods

Although the model is motivated by motifs relevant to MHC–peptide binding motifs, it is broadly applicable to other biological binding scenarios. Accordingly, the model is formulated in a general framework suitable for a wide range of biological sequence data.

### 2.1 Model

Let $R=\left(r_{1},r_{2},\ldots ,r_{n}\right)$ denote a collection of *n* observed biological sequences. We assume the existence of *K* latent motifs, each representing a distinct binding preference. Each sequence $r_{i}$ is assumed to either contain an instance of one of these motifs or to be generated entirely from a background distribution, without any motif. We introduce a vector of latent variables $W=(w_{1},w_{2},\ldots ,w_{n})$, where each $w_{i}\in \{1,2,\ldots ,K+1\}$ indicates the motif associated with sequence $r_{i}$. Specifically, $w_{i}=k$ (for 1≤k≤K) denotes that the *i*th sequence contains an instance of the *k*th motif, while $w_{i}=K+1$ implies that the sequence is purely drawn from the background distribution, without containing any known motif. We further denote the subset of unknown motif labels as $W_{U}=\{w_{u_{1}},w_{u_{2}},\ldots ,w_{u_{l}}\}$, and the known labels as $W_{U^{c}}=W\setminus W_{U}$.

Consider $A=\{A_{1},A_{2},\ldots ,A_{K}\}$ as the collection of binding location matrices. The *k*th matrix $A_{k}={\left[a_{\mathrm{ijk}}\right]}_{1\leq i\leq n,1\leq j\leq J_{k}}$ encodes the positions of the *k*th motif in sequences where $w_{i}=k$, with the motif length $J_{k}$ assumed to be known. Here, $a_{\mathrm{ijk}}$ represents the position of the *j*th element of the *k*th motif in the *i*th sequence. The values of $a_{\mathrm{ijk}}$ are constrained by the previous position $a_{i(j-1)k}$ to ensure a valid ordering of motif positions along the sequence. Specifically, for $2\leq j\leq J_{k}$, the allowed range for $a_{\mathrm{ijk}}$ is


$$a_{\mathrm{ijk}}\in \{a_{i(j-1)k}+1,a_{i(j-1)k}+2,\ldots ,L_{i}-J_{k}+j\},$$

where $L_{i}$ is the known length of sequence $r_{i}$. This condition ensures that the motif fits within the bounds of the sequence and respects the sequential order of positions. To model the expected spacing between motif positions, we place a prior distribution on the gap $a_{\mathrm{ijk}}-a_{i(j-1)k}$, assuming it follows a Poisson distribution with rate parameter $\lambda_{k(j-1)}$. The Poisson distribution, or its exponential counterpart, is commonly used to model motif spacing and indel lengths due to its simplicity and biological plausibility (Durbin *et al.* 1998, Frith *et al.* 2008). In our setting, the Poisson prior reflects a soft preference for certain gap lengths between conserved motif segments, which may correspond to structural constraints or selection for specific spacer lengths. Let $\Lambda =\{\lambda_{1},\lambda_{2},\ldots ,\lambda_{K}\}$ denote the set of Poisson parameters, where $\lambda_{k}={\left[\lambda_{k1},\lambda_{k2},\ldots ,\lambda_{k(J_{k}-1)}\right]}^{T}$ specifies the prior gap structure for the *k*th motif.

We assume that letters at motif-binding positions are generated independently from one of the *K* distinct product categorical distributions. Specifically, a product categorical distribution refers to a sequence of independent categorical distributions, one for each position within a motif. That is, for a motif of length $J_{k}$, each position $j\in \{1,\ldots ,J_{k}\}$ has its own categorical distribution over an alphabet of size *p*, and the letters at different positions are assumed to be drawn independently. Let $\vartheta =\{\Theta_{1},\Theta_{2},\ldots ,\Theta_{K}\}$ denote the set of position-specific probability matrices. Each matrix $\Theta_{k}={\left[\theta_{\mathrm{ijk}}\right]}_{1\leq i\leq p,1\leq j\leq J_{k}}$ defines the categorical probabilities for the *k*th motif, where $\theta_{\mathrm{ijk}}$ denotes the probability of observing the *i*th letter at position *j* in motif *k*. The *j*th column vector $\Theta_{jk}={\left[\theta_{1jk},\theta_{2jk},\ldots ,\theta_{\mathrm{pjk}}\right]}^{T}$ thus represents the categorical distribution at the *j*th binding position of motif *k*. Here, *p* is the size of the alphabet, which is typically p=4 for DNA sequences (A, C, G, T) and p=20 for amino acid sequences. For all positions not assigned to any motif, we assume the letters are independently drawn from a background categorical distribution with probability vector $\theta_{0}$.

Let h(·) denote the function that maps a sequence or subsequence to its count vector over the alphabet $\left\{O_{1},O_{2},\ldots ,O_{p}\right\}$. Specifically, for a sequence segment *s*, we define:


$$h(s)={\left[\sum_{x\in s}I(x=O_{1}),\sum_{x\in s}I(x=O_{2}),\ldots ,\sum_{x\in s}I(x=O_{p})\right]}^{T}.$$

Each sequence $r_{i}$ falls into one of the two possible scenarios:

1. No binding occurs ($w_{i}=K+1$): In this case, the entire sequence is assumed to be generated from the background distribution $\theta_{0}$. The likelihood for $r_{i}$ is $\theta_{0}^{h(r_{i})}$.
2. A motif binding occurs ($w_{i}=k$, where 1≤k≤K): In this case, sequence $r_{i}$ contains an instance of the *k*th motif at positions specified by $a_{i\cdot k}={\left[a_{\mathrm{ijk}}\right]}_{1\leq j\leq J_{k}}$, i.e. the *i*th row of the binding position matrix $A_{k}$. The residues at these binding positions are drawn independently from the corresponding product categorical distribution $\Theta_{k}$, while the remaining residues follow the background distribution $\theta_{0}$. The likelihood for $r_{i}$ in this case is
  $$\theta_{0}^{h(r_{i},{\left\{a_{i\cdot k}\right\}}^{c})}\prod_{j=1}^{J_{k}}\Theta_{\cdot jk}^{h(r_{i,a_{\mathrm{ijk}}})},$$

where ${\left\{a_{i\cdot k}\right\}}^{c}$ denotes the set of positions in $r_{i}$ excluding the motif-binding sites.

**Figure 3:**
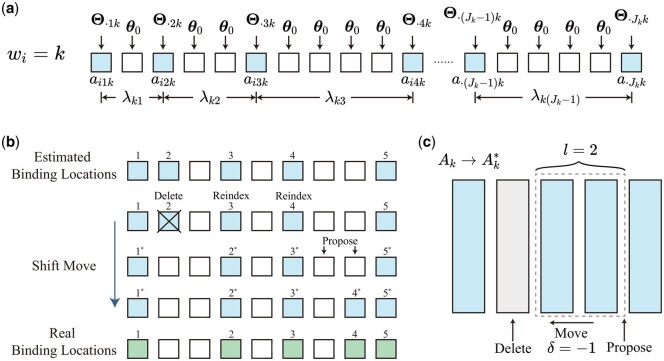
Model diagram and shift move. (a) The relationship between the parameters and the observed sequence. (b) A toy example illustrating the shift move used to escape from a local mode in the posterior. (c) The schematic representation of the shift move shown in panel (b), highlighting the three-step operation: delete, move, and propose.

The full likelihood of all observed sequences, given the known motif assignments $W_{U^{c}}$ and unknown ones $W_{U}$, is


$$\begin{array}{cc} & p\left(R|W_{U^{c}},W_{U},A,\vartheta ,\theta_{0},\Lambda \right)\\ \propto & \prod_{i=1}^{n}\prod_{k=1}^{K}{\left[\theta_{0}^{h(r_{i},{\left\{a_{i\cdot k}\right\}}^{c})}\prod_{j=1}^{J_{k}}\Theta_{\cdot jk}^{h(r_{i,a_{\mathrm{ijk}}})}\right]}^{I(w_{i}=k)}\times {\left[\theta_{0}^{h(r_{i})}\right]}^{I(w_{i}=K+1)}, \end{array}$$

where I(·) is the indicator function. Figure 3a presents a toy example that demonstrates the relationship between the parameters and the sequence.

The term $\Theta_{\cdot jk}^{h(r_{i,a_{\mathrm{ijk}}})}$ denotes the likelihood of observing letter $r_{i,a_{\mathrm{ijk}}}$ at the *j*th binding position of the *k*th motif. Let $\left\{O_{1},O_{2},\ldots ,O_{p}\right\}$ denote the set of possible letters in the alphabet. Then, this term can be written as


$$\Theta_{\cdot jk}^{h(r_{i,a_{\mathrm{ijk}}})}=\prod_{l=1}^{p}\theta_{\mathrm{ljk}}^{I(r_{i,a_{\mathrm{ijk}}}=O_{l})}.$$

The formulas for parameter updates based on MCMC and a full description of the algorithm are provided in the Supplementary Material, available as supplementary data at *Bioinformatics* online. The main computational burden arises from updating the motif position matrix A. Since positions are sampled sequentially within each row rather than enumerating the full configuration space, the per-iteration time complexity remains O(nL), equivalent to the gap-free case. A detailed complexity analysis is provided in the Supplementary Material, available as supplementary data at *Bioinformatics* online. To improve mixing in the more structured latent space introduced by gap-aware modeling, we incorporate a new shift move based on the Metropolis–Hastings (MH) algorithm, described in the next section.

### 2.2 Shift move to jump out of the local mode based on MH algorithm

As noted in the Gibbs sampling framework for motif discovery (Lawrence *et al.* 1993, Liu 1994), motif sampling procedures are prone to getting trapped in local modes. The analysis in that work assumes that motif-binding sites are contiguous and that each sequence contains at most one instance of a single motif type. Under these simplifying assumptions, the structure of local modes becomes relatively tractable and has been well characterized. However, our model includes multiple motif types, and the binding locations may not be consecutive. These irregularities introduce additional complexity into the posterior distribution, resulting in a more rugged landscape with diverse local modes that are more difficult to escape. Consequently, a modified shift move based on MH algorithm becomes necessary.

Let the true binding locations for the *k*th motif be represented by the matrix


$$A_{k}^{0}=\left[A_{1k}^{0},A_{2k}^{0},\ldots ,A_{J_{k}k}^{0}\right],$$

where each column vector $A_{jk}^{0}$ contains the positions of the *j*th site of the *k*th motif across all sequences. In practice, the sampler may converge to a mode


$$A_{k}^{*}=\left[A_{1k}^{*},A_{2k}^{*},\ldots ,A_{J_{k}k}^{*}\right],$$

where for a subset of indices $S\subseteq \{1,\ldots ,J_{k}\}$, the sampled positions satisfy


$$A_{jk}^{*}=A_{(j+\delta )k}^{0},\;\text{for}\;\text{some}\;\text{fixed}\;\delta ,\;j\in S.$$

In other words, the motif locations in the local mode are collectively shifted in index by a constant offset δ, relative to the true binding locations. Although these sampled positions do not correspond to the true motif locations, they may still partially align with conserved regions of the motif, particularly if the motif is short or exhibits a strong core pattern. This partial overlap can result in a relatively high posterior probability for $A_{k}^{*}$, making it a local mode. Due to the discreteness of the location space, escaping from such a local mode requires proposing coordinated changes to multiple columns of $A_{k}$. Standard sampling procedures have difficulty making such global moves, which leads to poor mixing and convergence around these misaligned modes.

Here we propose a shift move to jump out of this kind of local mode. Given the strong interdependencies among variables, particularly between the *k*th binding locations, the motif matrix $\Theta_{k}$, and the Poisson gap parameters $\lambda_{k}$, proposing updates to the binding locations alone often leads to low acceptance rates in the MH procedure. To mitigate this issue, we jointly propose updates to the binding locations $A_{k}$, the motif matrix $\Theta_{k}$, and the Poisson gap parameters $\lambda_{k}$ within an MH framework. In each MH proposal, we select a contiguous block of *l* columns within $A_{k}$ and attempt to shift this block by one position, either forward or backward. The shift creates a vacancy at one end of the block, which is filled by proposing a new column of binding locations. To ensure consistency, we simultaneously shift the corresponding motif columns in $\Theta_{k}$, and propose a new column for the vacated position from a posterior Dirichlet distribution based on the residues at the proposed binding sites. The gap parameters $\lambda_{k}$, which govern the spacing between adjacent motif positions, are proposed using a Gaussian random walk in log space. These coordinated proposals maintain alignment between the shifted binding sites, motif distributions, and spacing structure, thereby improving the acceptance rate and enabling the sampler to escape local modes. Further algorithmic details are provided in the Supplementary Material, available as supplementary data at *Bioinformatics* online.

Figure 3b and c provides a toy example illustrating how the shift move operates to escape from a local mode. In this example, the current estimate of the binding location matrix $A_{k}$ is misaligned with respect to the true binding positions, a typical manifestation of a local mode. In this example, we consider a shift move which includes a backward shift (δ=−1) applied to a block of length l=2. As shown in panel (b), the second estimated binding location are not in the true motif locations (green). The shift move proceeds by deleting the second column in the estimated binding matrix, shifting the selected block one position backward, and inserting a newly proposed column at the end of the block. This reconfiguration results in a new candidate matrix $A_{k}^{*}$, where the shifted columns are partially realigned with the true motif positions. Panel (c) presents a schematic view of this operation. This coordinated block-level shift allows the sampler to escape from the local mode by exploring alternate alignments of the motif across sequences.

## 3. Results

### 3.1 Simulation study

We conducted a series of simulation studies to evaluate the performance of the proposed algorithm (GAMMA). Synthetic datasets were generated using known parameter values, and the algorithm was then applied to estimate these parameters. Inference accuracy was assessed by comparing the estimated values to their ground-truth counterparts, with close agreement indicating effective performance.

To examine the algorithm’s robustness under varying levels of complexity, we designed two simulation scenarios. In the first scenario, all sequences were generated based on a single motif type. Each sequence either contained this motif or no motif at all. This setup enabled a focused evaluation of the algorithm’s sensitivity to detecting the presence or absence of a specific motif. In the second scenario, the dataset included multiple motif types distributed across sequences. Each sequence contained one of the multiple motif types or none. This setting introduced additional complexity, requiring the algorithm not only to detect motif presence but also to accurately identify which motif type was embedded in each sequence. Together, these two scenarios allowed us to assess both the detection sensitivity and the discriminative capability of the algorithm under progressively more challenging conditions.

#### 3.1.1 Single-motif detection

We consider the single-motif scenario. In this setting, we generated n=200 biological sequences, each of length 10. The background parameters $\theta_{0}$ was sampled from a symmetric Dirichlet prior, Dirichlet(1). The motif length was set to $J_{1}$. For each position $j=1,\ldots ,J_{1}$, the motif matrix $\Theta_{j1}$ was drawn from a Dirichlet distribution with concentration parameter η. The motif-binding label for each sequence was drawn from a categorical distribution Cat(0.8,0.2), where $w_{i}=1$ indicates the presence of the motif and $w_{i}=2$ indicates its absence. For sequences with $w_{i}=1$, the initial motif-binding site $a_{i11}$ was sampled uniformly at random. To model the spacing between motif-binding sites, the Poisson parameter $\lambda_{1j}$ was sampled randomly from a predefined set *S*. Subsequent motif-binding positions $a_{ij1}$ for $j=2,\ldots ,J_{1}$ were generated from a truncated Poisson distribution, $\mathrm{Poi}s_{+}(\lambda_{1j},a_{i(j-1)1})$, conditioned on the previous site. The complete collection of motif-binding locations for the single-motif setting is denoted by the matrix $A_{1}$.

To evaluate the algorithm’s performance under varying conditions, we explored different combinations of motif length ($J_{1}$), the Dirichlet concentration parameter η, and the parameter set *S* from which the Poisson rates $\lambda_{1j}$ were sampled. Specifically, the motif length $J_{1}$ was set to either 4 or 5. The Dirichlet parameter η, which controls the concentration of the motif distribution, was selected from the set {0.1,0.2}. The Poisson parameters $\lambda_{1j}$ were drawn from one of the two sets S∈{{0.5,1.0,1.5}, {1.5,2.0,2.5}}, representing different motif spacing scenarios.

**Figure 4:**
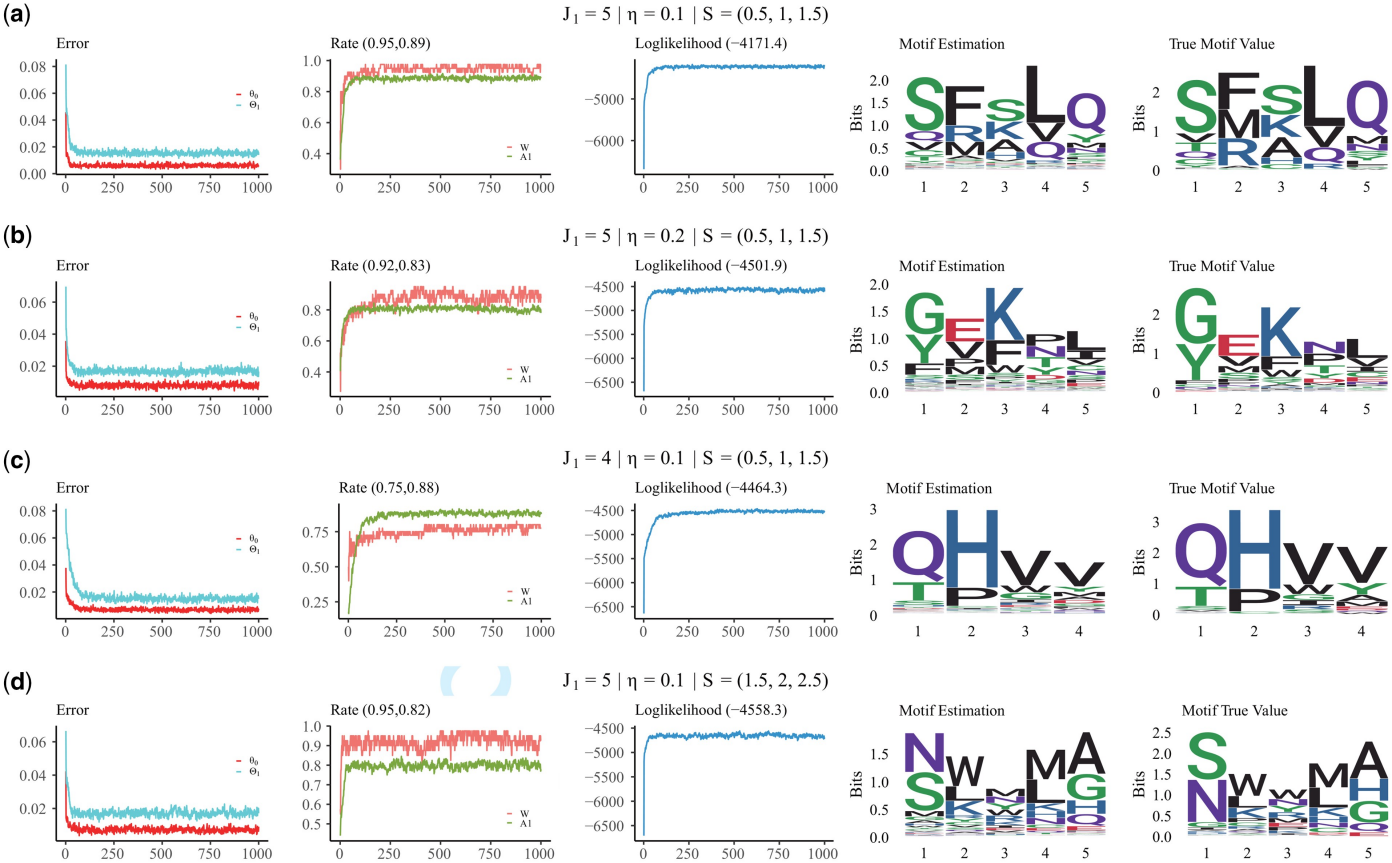
Inference performance under a single-motif simulation scenario. Subfigures (a) to (d) correspond to four different simulated datasets under varying settings. In each subfigure, the first column presents trace plots of errors, where red and blue curves correspond to $\theta_{0}$ and $\Theta_{1}$, respectively. The second column shows accuracy trace plots for W (orange) and $A_{1}$ (green). The third column illustrates the log-likelihood trace plots. The fourth and fifth columns display sequence logos (created by ggseqlogo; Wagih 2017): estimated and true $\Theta_{1}$, respectively.

The motif length $J_{1}$ is varied to assess the algorithm’s sensitivity to different motif sizes. Shorter motifs are generally more difficult to detect due to their limited information content, which makes them harder to distinguish from background sequences. In contrast, longer motifs provide more sequence context that can aid in detection, but they also increase the search space and computational complexity. Motif conservation is controlled by the Dirichlet concentration parameter η. Smaller values (e.g. 0.1) produce sharply peaked amino acid distributions, representing highly conserved motifs dominated by a few residues. Larger values (e.g. 0.2) result in flatter distributions, indicating more diverse and less conserved motifs. The parameter $\lambda_{1j}$ of the gap distribution is drawn from the parameter set *S*, which determines the expected distance between consecutive motifs. Lower values of $\lambda_{1j}$ (e.g. S={0.5,1.0,1.5}) simulate densely packed motif positions with shorter intersite distances, while higher values (e.g. S={1.5,2.0,2.5}) represent sparsely distributed motifs with longer gaps between sites. The specific simulation settings, including motif length $J_{1}$, Dirichlet parameter η, and Poisson parameter set *S*, are indicated in the titles of the subfigures in Fig. 4.

Additionally, to reflect the presence of missing or unobservable data in realistic biological conditions, we introduced label masking in W, where a proportion of the labels were set to “NA.” Specifically, 20% of the $w_{i}$ values were masked. This setting mimics practical limitations in experimental data collection.

We implemented the MCMC algorithm using noninformative (uniform) priors for the parameters and latent variables $\theta_{0}$, $\Theta_{j1}$, *W*, and $a_{i11}$, assigning equal prior probability to all admissible values within their respective domains. These noninformative prior choices are intended to minimize the influence of subjective assumptions and ensure that posterior inference is primarily driven by the observed data. For the Poisson parameter $\lambda_{1j}$, we specified a Gamma prior with shape and rate parameters $\beta_{1j}=\nu_{1j}=1$, which corresponds to an exponential distribution with a mean of 1. A total of 1000 iterations were performed using the MCMC sampler, with the first 500 iterations discarded as burn-in to ensure convergence. Initial values for all model parameters and latent variables were drawn from their respective prior distributions.

Algorithm performance is evaluated through several key metrics. The error curves for $\theta_{0}$ and $\Theta_{1}$ illustrate the normalized L1-norm of the absolute errors over time. The normalized $\ell_{1}$-norm quantifies the average absolute error, computed as the total absolute error divided by the number of elements. The accuracy curves for the latent variables W and $A_{1}$ represent the proportion of correct predictions. The loglikelihood curve, which plots log-likelihood values across iterations, serves as an indicator of model fit, where stability and an upward trend suggest good convergence. Additionally, sequence logos of the estimated motifs compared to the true motifs provide a visual assessment of motif estimation accuracy.

The corresponding simulation results are shown in Fig. 4. Each experimental condition is characterized by a tuple $\left(J_{1},\eta ,S\right)$, where $J_{1}$ denotes the motif length, η is the Dirichlet concentration parameter used for generating $\Theta_{j1}$, and *S* represents the set from which the Poisson parameter $\lambda_{1j}$ is sampled. In the subfigures, the values displayed after the title *Rate* denote the accuracy of the maximum a posteriori (MAP) estimates, whereas those following *Loglikelihood* indicate the corresponding likelihood values. The results show that the algorithm exhibits robust performance across various combinations of motif length, conservation level, and binding site spacing. The high accuracy of *W* indicates that the algorithm performs well in determining whether the motif is present in each sequence. In addition, the accuracy of $A_{1}$, along with the close match between the estimated motif and the ground truth, demonstrates that the algorithm is also effective in identifying the motif positions and recovering the underlying motif pattern.

Several trends emerge from the results. From Fig. 4a and b, we observe that as the motif becomes less conserved (i.e. higher entropy due to a larger Dirichlet parameter η), the estimation accuracy for both the binding label *W* and the binding location $A_{1}$ declines. This is expected, as weaker motif signals introduce more ambiguity in distinguishing motif-containing sequences and localizing the binding sites. Similarly, comparing Fig. 4a and c, we find that reducing the motif length $J_{1}$ while keeping the total sequence length fixed leads to a lower signal-to-noise ratio. This results in a noticeable drop in the accuracy of both *W* and $A_{1}$, consistent with the intuition that shorter motifs are harder to detect reliably in longer background sequences. Finally, Fig. 4d shows that increasing the spacing between binding sites (i.e. sampling $\lambda_{1j}$ from a distribution with larger support) leads to decreased accuracy in estimating $A_{1}$, even though the accuracy of *W* remains high. This suggests that while the presence of the motif can still be detected reliably, the precise localization of the binding site becomes more challenging when the motif instances are more sparsely distributed. These observed patterns are consistent with theoretical expectations and highlight the sensitivity of motif discovery accuracy to motif strength, length, and spatial density. Additional details and quantitative metrics for each simulation setting are provided in the Supplementary Material, available as supplementary data at *Bioinformatics* online.

**Figure 5:**
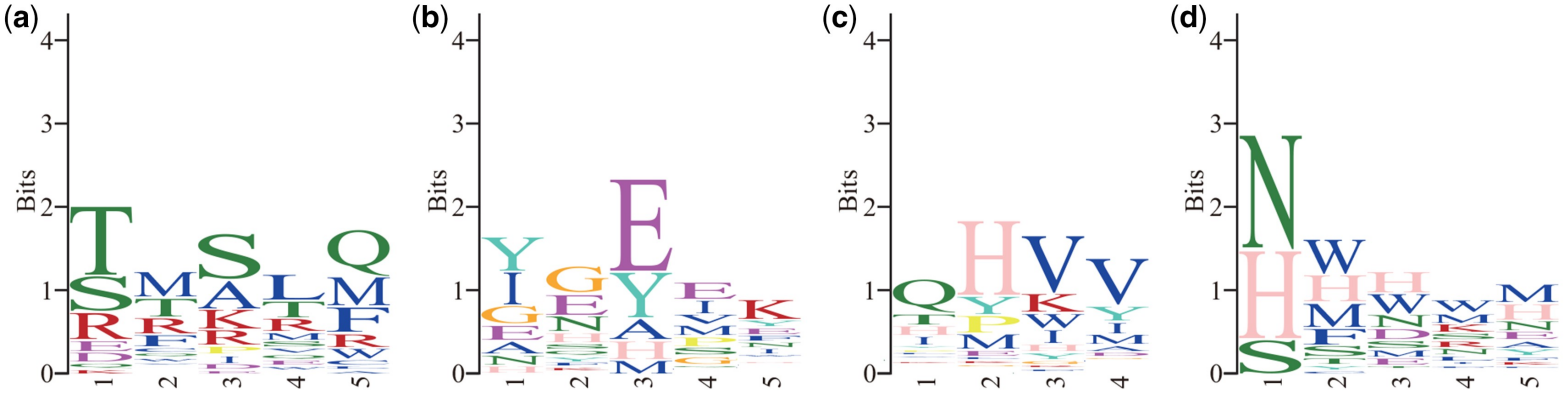
Motif estimated by GLAM2 on simulated data. Subfigures (a) to (d) show the results of the existing algorithm GLAM2 on four different simulated datasets, corresponding to the same settings as in Fig. 4, for comparison with our proposed method.

For comparison, we applied the existing motif discovery algorithm GLAM2 (Frith *et al.* 2008) to the same simulated datasets (Fig. 5). GLAM2 was configured to perform up to 1000 iterations per run, with the motif length fixed to the true motif length. As shown in the figure, except for the third scenario, the motifs estimated by GLAM2 deviate substantially from the ground truth. In contrast, our proposed method produces estimates that are closely aligned with the true motifs, as illustrated in Fig. 4. Even in the third scenario, our method yields more accurate results than GLAM2. To quantitatively evaluate motif accuracy, we computed the Kullback–Leibler (KL) divergence between the true motif and the motifs estimated by each method. Results from four simulation settings show that GAMMA consistently produces estimates with substantially lower KL divergence compared to GLAM2 (Table 1), indicating closer alignment with the true underlying motif. These findings provide strong quantitative support for the effectiveness of GAMMA. Further simulation results for the multimotif detection case are provided in the Supplementary Material, available as supplementary data at *Bioinformatics* online. We also include separate analyses of how the block length and frequency of shift moves affect convergence and sampling efficiency, as well as how correlations between model parameters influence motif inference performance.

**Table 1:** KL divergence between estimated motifs and the ground truth across four simulation settings.

ID	Simulation setting	KL (GAMMA)	KL (GLAM2)
(1)	$J_{1}=5\;|\;\eta =0.1\;|\;S=(0.5,\;1,\;1.5)$	0.444	2.982
(2)	$J_{1}=5\;|\;\eta =0.2\;|\;S=(0.5,\;1,\;1.5)$	0.193	2.983
(3)	$J_{1}=4\;|\;\eta =0.1\;|\;S=(0.5,\;1,\;1.5)$	0.492	2.695
(4)	$J_{1}=5\;|\;\eta =0.1\;|\;S=(1.5,\;2,\;2.5)$	0.301	4.265

GAMMA consistently yields lower divergence than GLAM2.

### 3.2 Real applications

We retrieved the MHC-bound peptide dataset from the MHC Motif Atlas (Tadros *et al.* 2023) and focused on three of the most common HLA Class I alleles in the Chinese population: HLA-A*02:01, HLA-A*24:02, and HLA-A*11:01. The corresponding motif logos and peptide length distributions are shown in Figs 1 and 2.

We applied our model separately to the dataset of each allele. Following the approach used in the simulation study, we randomly masked 20% of the peptide labels and used our algorithm to predict the masked labels. To determine the most appropriate motif length for each allele, we tested motif lengths ranging from 2 to 13. Motif length 14 was excluded due to the small number of peptides of that length. For each setting, we recorded the prediction accuracy on the masked labels. The results are summarized in Table 2.

**Table 2:** Prediction accuracy of peptide binding for different HLA-A alleles across various motif lengths. Bold values indicate the highest prediction accuracy for each HLA-A allele across motif lengths.

HLA allele	Motif length											
	2	3	4	5	6	7	8	9	10	11	12	13
HLA-A*02:01	0.693	0.741	0.792	0.784	0.827	0.859	**0.931**	0.858	0.814	0.819	0.803	0.830
HLA-A*11:01	0.849	0.871	0.879	0.890	0.909	0.904	**0.917**	0.904	0.901	0.873	0.882	0.893
HLA-A*24:02	0.742	0.779	0.819	0.864	0.862	0.884	**0.913**	0.889	0.894	0.908	0.839	0.810

From the figure, we observe that the prediction accuracy reaches its peak when the motif length is set to 8 for all three alleles. Initially, we hypothesized that a motif length of 2 would be optimal, as only one or two positions appear to be strongly conserved in the sequence logos from the MHC Motif Atlas (Fig. 1). However, the accuracy values at length 2, 0.693 for HLA-A*02:01, 0.849 for HLA-A*24:02, and 0.742 for HLA-A*11:01, are notably lower than those at length 8. This suggests that additional positions beyond the most conserved sites contribute meaningfully to peptide binding.

**Figure 6:**
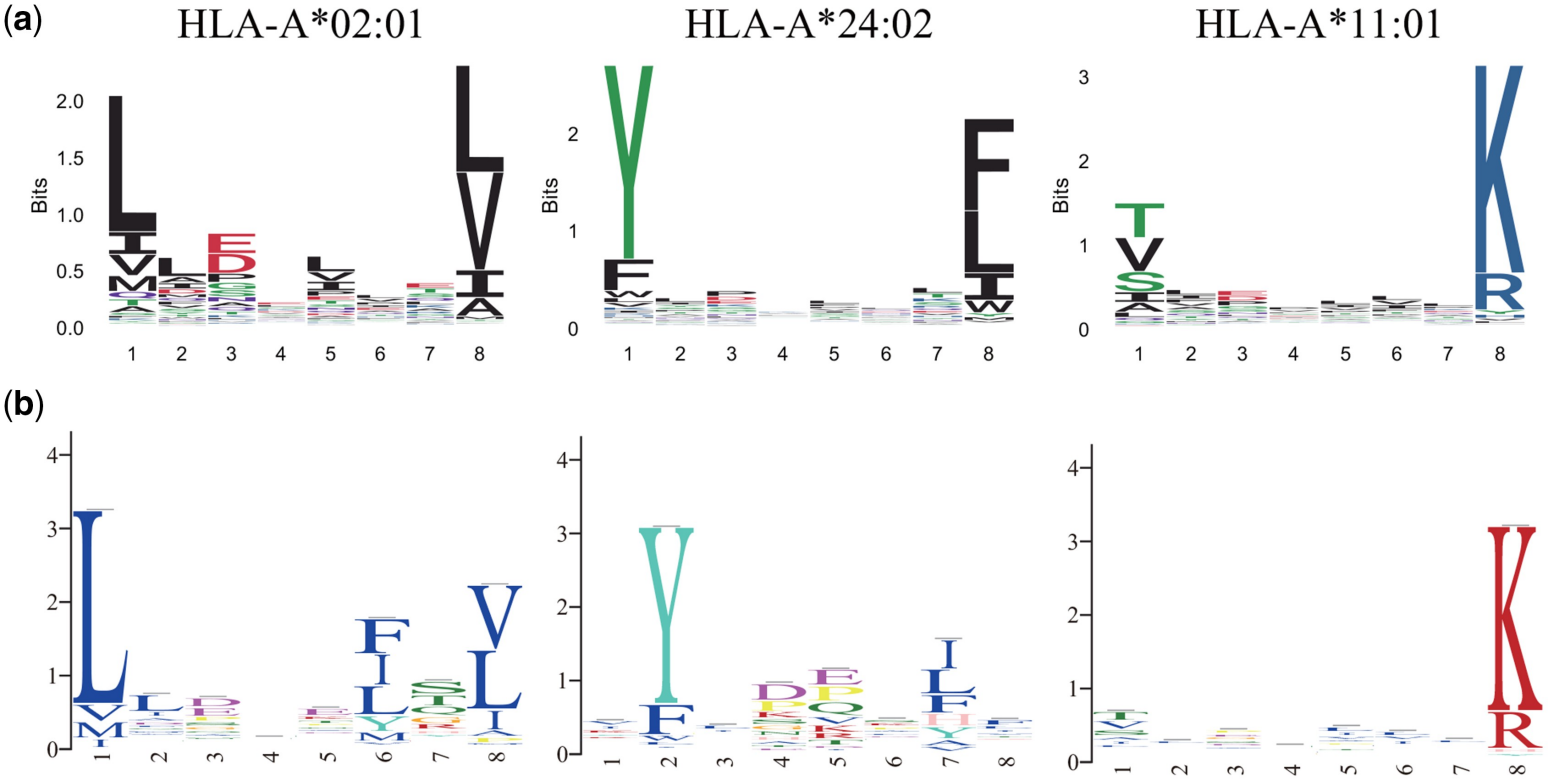
Sequence logos for the estimated motifs. (a) The first row represents the sequence logos for the motifs estimated by our method. (b) The second row represents the sequence logos for the motifs estimated by GLAM2.

Notably, the optimal length of 8 differs slightly from the widely held assumption that MHC Class I motifs typically span nine binding positions. To further investigate this discrepancy, we visualized the sequence logos of the estimated motifs under the optimal motif length (Fig. 6a). Compared to the logos from the MHC Motif Atlas, our model assigns relatively low importance to the first position of the canonical nine-residue motif. In contrast, both the second and final positions remain highly conserved, although the position corresponding to the second binding residue in the traditional motif appears to shift to the first position in our model. Structural evidence from the PDB (e.g. 1HHG-1HHK, 7N1A-7N1F, 3RL1-3RL2) further supports this observation (Madden *et al.* 1993, Zhang *et al.* 2011, Wu *et al.* 2022). In crystal structures of nine-mer peptides bound to MHC Class I molecules, the first residue is not buried in the binding groove, whereas the second and ninth residues act as primary anchor points. These observations align with our model’s result that eight residues, excluding the first, are sufficient to capture the core binding motif.

For comparison, we applied GLAM2 to estimate the underlying motifs, with the motif length fixed at 8. The resulting sequence logos are shown in Fig. 6b. For HLA-A*02:01, the motif inferred by GLAM2 displays a highly conserved column at the sixth position, which is not observed in the sequence logos from the MHC Motif Atlas. Similarly, for HLA-A*24:02, GLAM2 identifies a strongly conserved site at the seventh position, but the corresponding amino acids differ from those in the MHC Motif Atlas. For HLA-A*11:01, the sequence logos generated by GLAM2, our method, and the MHC Motif Atlas are similar. Overall, these results indicate that our method provides more biologically plausible motif estimates than GLAM2, particularly for HLA-A*02:01 and HLA-A*24:02.

In addition to GLAM2, we further compared GAMMA with two widely adopted MHC binding prediction tools: NetMHCpan-4.1 and MixMHCpred-3.0. To ensure a consistent evaluation, we used the same dataset where 20% of the peptide binding labels were randomly masked. Each method was applied to predict the binding status of the masked peptides, and prediction accuracy was calculated by comparing the predicted labels to the ground truth. Both NetMHCpan-4.1 and MixMHCpred-3.0 were run using their default parameters, as commonly done in practice. The results are presented in Table 3. Across all three HLA alleles, GAMMA achieved comparable or higher accuracy compared to NetMHCpan and MixMHCpred. While the latter tools are known for their predictive power, they do not return interpretable motif structures directly. In contrast, GAMMA not only performs competitively in prediction but also uncovers biologically meaningful motifs, such as noncontiguous residue patterns and spacing flexibility.

**Table 3:** Prediction accuracy on masked peptide labels for three HLA alleles using different methods.

Method	HLA-A*02:01	HLA-A*11:01	HLA-A*24:02
GAMMA (ours)	0.931	0.917	0.913
NetMHCpan-4.1	0.806	0.831	0.912
MixMHCpred-3.0	0.872	0.884	0.944

**Figure 7:**
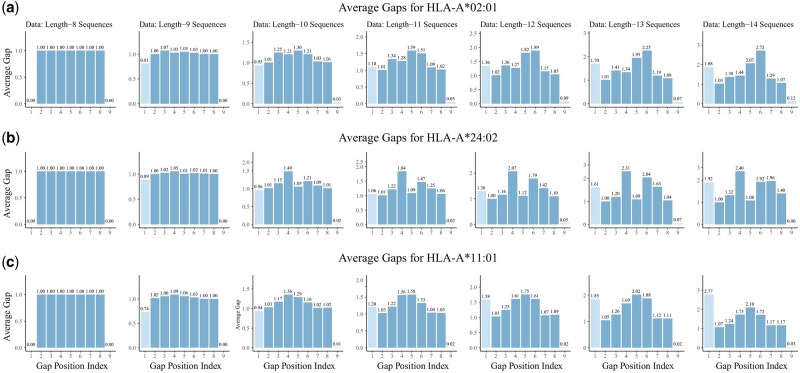
Distribution of average gaps between motif and flanking regions. Subfigures (a) to (c) show the average distances between adjacent positions in an extended motif position matrix (200 × 10) for three different HLA-A alleles. In each subfigure, the first column represents the start of each sequence (fixed at position 1), the last column represents the end position of each sequence, and the eight columns in between correspond to motif-binding positions. The *y*-axis shows the average gap length between each adjacent pair of positions, while the *x*-axis indicates the index of each gap segment from start to end.

To further investigate the spacing patterns between motif positions under the optimal motif length, we estimated the binding positions for each sequence and computed the distances between adjacent binding sites. In addition, we calculated the distance from the start of the sequence to the first binding position, as well as the distance from the last binding position to the end of the sequence. Together, this yields nine gap values per sequence: one flanking gap at each end and seven internal gaps between binding positions. We then computed the average of each of these nine gap values across sequences of different lengths. The results are shown in Fig. 7, where each gap index corresponds to a specific segment: from the first sequence position to the first binding site, between consecutive binding sites, and from the last binding site to the final sequence position.

As shown in the figure, the three alleles exhibit similar gap distribution patterns. In the first column, corresponding to peptides of length 8, all eight residues are used as binding positions. Consequently, the internal gaps between binding sites are all equal to one, and the flanking gaps at both ends are zero, since the motif spans the entire sequence. As sequence length increases, the gap between the last binding position and the end of the sequence remains close to zero across all alleles, suggesting a strong preference for the final residue to serve as a binding site. The second internal gap consistently averages around one, indicating that the second binding position is typically adjacent to the first. In contrast, the average gap between the start of the sequence and the first binding position increases with sequence length, implying that upstream residues are more likely to be excluded from the motif as peptide length grows.

As peptide length increases, all three alleles tend to expand spacing in the central region of the peptide, resulting in a consistent “central bulging” pattern. This effect is especially pronounced starting from peptide length 10 and above, where internal gaps near the middle positions gradually increase. We also performed a sensitivity analysis using different hyperparameters for the Gamma prior on the Poisson rate in the Supplementary Material, available as supplementary data at *Bioinformatics* online, and found that the central bulging effect remains robust across a range of prior settings.

To statistically validate this observation, we performed a nonparametric permutation test using peptides of length 9–14. For each allele, we compared the average gap values between positions P4–P6 against those in the other regions. To generate an empirical null distribution, we randomly shuffled the gap values within each individual peptide sequence, thereby preserving the independence of each sequence. Under the null hypothesis that all gap positions are exchangeable and there is no systematic difference between central and noncentral gaps, the observed difference in mean gap values should follow this empirical null distribution. However, in our analysis, none of the 100 000 permutations produced a difference greater than or equal to the observed value for any allele, indicating a highly significant and robust central bulging effect (empirical $p<1\times 10^{-5}$).

This statistically supported pattern reflects a structural preference for central bulging of the peptide within the MHC binding groove, with the N-terminal flanking region more likely to remain unbound. MHC Class I molecules typically anchor peptides at their termini, while longer peptides tend to adopt a bulged conformation centrally to accommodate the closed binding groove. Central positions, especially P4–P6, are more prone to exhibit bulging or increased spacing as peptide length increases, whereas N-terminal residues often extend beyond the binding site or remain unanchored (Tynan *et al.* 2007).

## 4. Discussion

In this work, we proposed GAMMA, a probabilistic framework designed to identify noncontiguous binding motifs under partially labeled data settings. Unlike traditional motif models for MHC binding that assume fixed-length contiguous binding cores, GAMMA allows flexible positioning of conserved residues with variable-length gaps between them. The model utilizes Bayesian inference with MCMC sampling, incorporating both Gibbs sampling and MH steps to efficiently explore the complex posterior landscape. Through both simulation studies and real-world MHC Class I peptide binding datasets (from the MHC Motif Atlas), GAMMA successfully identified the binding positions and demonstrated superior performance compared to existing motif discovery tools such as GLAM2.

A key finding of our study is that, contrary to the widely accepted belief that MHC Class I binding motifs span nine positions, with the second and last positions being particularly conserved, our model suggests that the true number of important positions may be only 8, with the first and last positions being most conserved. This deviation from conventional understanding is interesting and invites further investigation. Notably, our results reveal that intermediate positions may include gaps, indicating that the binding residues are not necessarily adjacent, and that the central region of the peptide may exhibit substantial structural flexibility.

This revised view of motif feature merits deeper biological and computational exploration. One promising direction is to validate whether this “8-position with gaps” motif pattern persists under alternative modeling paradigms, such as neural network-based methods. For instance, adapting NNalign (Nielsen and Lund 2009) or other deep learning-based frameworks with gap-awareness could provide an independent confirmation of our findings or offer new insights into the motif feature. If similar results are observed across modeling approaches, it would strengthen the hypothesis that current nine-position assumptions may oversimplify the true binding dynamics of MHC–peptide interactions.

Another possible future direction is the development of a simple and interpretable nonneural function that maps estimated binding positions to binding affinity. While deep learning models often offer high predictive accuracy, they may lack interpretability. In a previous study (Liu *et al.* 2023), we investigated this direction by building a linear regression model that relates motif features extracted from contiguous motifs to binding strength. However, that work did not account for gaps between binding positions, potentially compromising the accuracy of motif identification, as demonstrated in the current study. Extending that approach to incorporate gap-aware motif features could lead to a compact mathematical formulation that improves both predictive performance and biological insight. Such a function would be valuable for hypothesis generation, experimental validation, and practical applications in settings with limited data or computational resources.

Our model currently treats observed positive labels as fixed and fully accurate. While this is consistent with most prior work in MHC–peptide analysis, in practice there may be false positives due to experimental noise. An interesting extension would be to model the true label $w_{i}$ for each sequence as a latent variable, and treat the observed label $y_{i}$ as prior information, specifying a conditional prior $p(w_{i}|y_{i})$. This would allow all $w_{i}$ to be inferred during MCMC, incorporating both the sequence-level likelihood and prior evidence from the observed label. Such a formulation could improve robustness to label noise, but may introduce new challenges. For instance, low-likelihood true positives might be misclassified as background, potentially distorting motif inference. We leave a full investigation of this approach to future work.

Our current evaluation focused on three of the most prevalent human MHC Class I alleles in the Chinese population: HLA-A*02:01, HLA-A*11:01, and HLA-A*24:02. While these alleles are highly representative, broader validation across additional MHC-I alleles, as well as MHC Class II molecules, is necessary to fully assess the generalizability of our approach. To explore the potential applicability of our method beyond the MHC-I setting, we conducted preliminary experiments on both MHC-II peptide binding data and transcription factor binding sites from the JASPAR database (Rauluseviciute *et al.* 2024). These early results, presented in the Supplementary Material, available as supplementary data at *Bioinformatics* online, suggest that our gap-aware framework can recover meaningful motif patterns in both longer peptide sequences and DNA regulatory elements. However, more rigorous investigation is warranted. In particular, MHC-II binding involves more flexible core positions and longer peptides, while DNA sequence motifs may exhibit different structural characteristics. We plan to adapt and extend our model to better accommodate these complexities in future work.

## Supplementary Material

btag014_Supplementary_Data

## Data Availability

The raw data and the source codes are available on GitHub (https://github.com/RanLIUaca/GAMMAmotif).
