# Supplementary material for: GAMMA: gap-aware motif mining under incomplete labeling with applications to MHC motifs
Source: Bioinformatics. 2026 Jan 14;42(2):btag014. doi: 10.1093/bioinformatics/btag014 (PMC12866627; doi:10.1093/bioinformatics/btag014)
Supplement: btag014_Supplementary_Data [file btag014_supplementary_data.pdf]

Supplementary Materials for
"GAMMA: Gap-aware Motif Mining under Incomplete Labeling with Applications to MHC Motifs"

1. Bayesian inference

We performed Bayesian inference using MCMC methods, which generate samples from the posterior distribution via a Markov chain whose stationary distribution corresponds to the target posterior. The sampling process begins with an initial distribution and, through iterative transitions, converges to the desired posterior. In this study, we employed two standard MCMC algorithms, Gibbs sampling and the Metropolis-Hastings (MH) algorithm, to sample both model parameters and latent variables. To facilitate efficient inference, conjugate priors were assigned to the unknown parameters:

$$\boldsymbol{\theta}_0 \sim Dirichlet\left(\boldsymbol{\alpha}_0\right), w_{u_i} \sim Cat\left(p_0\right),\ 1 \le i \le l,$$
$$\boldsymbol{\Theta}_{\cdot jk} \sim Dirichlet\left(\boldsymbol{\alpha}_{jk}\right),\ 1 \le j \le J_k, 1 \le k \le K\ ,$$
$$\lambda_{kj} \sim Gamma(\beta_{kj}, \nu_{kj}), \quad 1 \le j \le J_k - 1, 1 \le k \le K.$$

The prior distributions of the latent binding positions in $\mathscr{A}$, conditional on the motif assignment vector $\boldsymbol{W}$, are specified as follows. The first binding position a_{i1k} is assumed to follow a categorical distribution over all valid starting positions, while subsequent positions a_{ijk} (for $j \ge 2$) follow a truncated Poisson distribution conditioned on the previous position:

$$a_{i1k} \sim \mathrm{Cat}(L_i - J_k + 1,\ \boldsymbol{\pi}_{0,a_i}), \quad 1 \le i \le n,\ 1 \le k \le K,$$
$$a_{ijk} \mid a_{i(j-1)k},\ w_i = k,\ \lambda_{k(j-1)}$$
$$\sim \mathrm{Pois}_+\left(\lambda_{k(j-1)};\ a_{i(j-1)k},\ L_i - J_k + j\right),$$
$$\text{for } 1 \le i \le n,\ 2 \le j \le J_k,\ 1 \le k \le K.$$

Here, $\mathrm{Cat}(L_i - J_k + 1,\ \boldsymbol{\pi}_{0,a_i})$ denotes a categorical distribution over the $L_i - J_k + 1$ valid starting positions, with probability vector $\boldsymbol{\pi}_{0,a_i}$. The distribution $\mathrm{Pois}_+(\lambda;\ a_{\mathrm{prev}},\ a_{\mathrm{max}})$ indicates a Poisson

distribution with rate λ, truncated to the integer interval $\{a_{\text{prev}}+1, \ldots, a_{\max}\}$, ensuring that motif positions remain ordered and within sequence bounds.

The truncated Poisson distribution used in the prior for a_{ijk} is defined as:

$$p\left(a_{ijk} \mid a_{i(j-1)k},\, w_i = k,\, \lambda_{k(j-1)}\right) = \frac{\dfrac{\lambda_{k(j-1)}^{a_{ijk}-a_{i(j-1)k}-1} e^{-\lambda_{k(j-1)}}}{\left(a_{ijk} - a_{i(j-1)k} - 1\right)!}}{\sum\limits_{x=0}^{L_i - J_k + j - a_{i(j-1)k} - 1} \dfrac{\lambda_{k(j-1)}^{x} e^{-\lambda_{k(j-1)}}}{x!}},$$

for $1 \leq i \leq n$, $2 \leq j \leq J_k$, and $1 \leq k \leq K$. This defines a Poisson distribution over the gap $a_{ijk} - a_{i(j-1)k} \in \{1, 2, \ldots, L_i - J_k + j - a_{i(j-1)k}\}$, normalized to ensure valid motif positioning within the sequence. The denominator serves as the normalizing constant to ensure the truncated distribution integrates to 1 over the allowable support.

Next, we derive the full conditional distributions for each parameter. For the motif-specific parameters $\boldsymbol{\Theta}_{\cdot jk}$ and the background parameter $\boldsymbol{\theta}_0$, the full conditionals are given by:

$$\boldsymbol{\Theta}_{\cdot jk} \mid - \sim \text{Dirichlet}\left(\boldsymbol{H}_{\boldsymbol{A}_{jk}} + \boldsymbol{\alpha}_{jk}\right), \quad 1 \leq j \leq J_k,\ 1 \leq k \leq K,$$
$$\boldsymbol{\theta}_0 \mid - \sim \text{Dirichlet}\left(\boldsymbol{H}_0 + \boldsymbol{\alpha}_0\right),$$

where $\boldsymbol{H}_{\boldsymbol{A}_{jk}}$ and $\boldsymbol{H}_0$ are defined as:

$$\boldsymbol{H}_{\boldsymbol{A}_{jk}} = \sum_{i=1}^{n} \mathbb{I}(w_i = k) \cdot h(r_{i,a_{ijk}}),$$
$$\boldsymbol{H}_0 = \sum_{i=1}^{n} \sum_{k=1}^{K} \mathbb{I}(w_i = k) \cdot h\left(r_{i,\{\boldsymbol{a}_{i\cdot k}\}^c}\right) + \sum_{i=1}^{n} \mathbb{I}(w_i = K+1) \cdot h(\boldsymbol{r}_i).$$

Here, $h(\cdot)$ denotes the count vector of letters over the alphabet, as previously defined. The notation '$-$' indicates conditioning on all other parameters, including the label vector $\boldsymbol{W}$, binding site positions $\mathscr{A}$, and observed sequence data $\boldsymbol{R}$.

For the binding label vector $\boldsymbol{W}$, the full conditional posterior distribution is:

$$w_{u_i} \mid - \sim \text{Cat}(p_{\text{pos}}), \quad 1 \leq i \leq l,$$

where the subscript 'pos' indicates posterior-related quantities, and $p_{\text{pos}} = \{p_{\text{pos},1}, p_{\text{pos},2}, \ldots, p_{\text{pos},K+1}\}$

is the normalized posterior probability vector. Each component $p_{\text{pos},k}$ is defined as

$$p_{\text{pos},k} = \frac{f_w(w_{u_i} = k)}{\sum_{l=1}^{K+1} f_w(w_{u_i} = l)},$$

where the unnormalized posterior weight $f_w(w_{u_i} = k)$ is given by

$$f_w(w_{u_i} = k) \triangleq \left[\boldsymbol{\theta}_0^{h(r_{u_i}, \{\boldsymbol{a}_{u_i \cdot k}\}^c)} \prod_{j=1}^{J_k} \boldsymbol{\Theta}_{\cdot jk}^{h(r_{u_i}, a_{u_i jk})} \right]^{\mathbb{I}(w_{u_i}=k)} \times \left[\boldsymbol{\theta}_0^{h(r_{u_i})} \right]^{\mathbb{I}(w_{u_i}=K+1)} \times p_{0k}^{\mathbb{I}(w_{u_i}=k)}.$$

For the binding location matrices $\boldsymbol{A}_k$, each row is conditionally independent given all other variables, allowing for parallel updates. The full conditional posterior distribution for each entry a_{ijk} is:

$$a_{ijk} \mid - \sim \text{Cat}(\boldsymbol{\pi}_{\text{pos},a_{ijk}}), \quad 1 \leq i \leq n,$$

where the subscript 'pos' indicates posterior-related quantities, and

$$\boldsymbol{\pi}_{\text{pos},a_{ijk}} = \left\{ \pi_{\text{pos},a_{ijk},a_{i(j-1)k}+1},\ \pi_{\text{pos},a_{ijk},a_{i(j-1)k}+2},\ \ldots, \pi_{\text{pos},a_{ijk},a_{i(j+1)k}-2},\ \pi_{\text{pos},a_{ijk},a_{i(j+1)k}-1} \right\}$$

is the normalized posterior probability vector over all valid candidate positions between $a_{i(j-1)k}$ and $a_{i(j+1)k}$. Each component $\pi_{\text{pos},a_{ijk},x}$ is defined as

$$\pi_{\text{pos},a_{ijk},x} = \frac{f_a(a_{ijk} = x)}{\sum_{y=a_{i(j-1)k}+1}^{a_{i(j+1)k}-1} f_a(a_{ijk} = y)},$$

where the unnormalized posterior weight $f_a(a_{ijk} = x)$ is given by

$$f_a(a_{ijk} = x) \triangleq \left[\boldsymbol{\theta}_0^{h(r_i, \{\boldsymbol{a}_{i \cdot k}(j,x)\}^c)} \boldsymbol{\Theta}_{\cdot jk}^{h(r_i, x)} \right]^{I(w_i=k)} \left[\frac{\lambda_{k(j-1)}^{x-a_{i(j-1)k}-1}}{(x - a_{i(j-1)k} - 1)!} \right]^{I(w_i=k, 2 \leq j \leq J_k)}$$
$$\times \left[\frac{\lambda_{kj}^{a_{i(j+1)k}-x-1}}{(a_{i(j+1)k} - x - 1)!} \right]^{I(w_i=k, 1 \leq j \leq J_k-1)} \Bigg/ \left[\sum_{y=0}^{L_i - J_k + (j+1) - x - 1} \frac{\lambda_{kj}^{y} e^{-\lambda_{kj}}}{y!} \right]^{I(w_i=k, 1 \leq j \leq J_k-1)}$$

where $a_{i(j-1)k} + 1 \leq x \leq a_{i(j+1)k} - 1$, and $\boldsymbol{a}_{i \cdot k}(j, x)$ denotes the vector $\boldsymbol{a}_{i \cdot k}$ with the j-th element replaced by x.

For the parameter λ_{kj}, the full conditional posterior distribution is given by:

$$p(\lambda_{kj} \mid -) \propto \frac{\prod_{i=1}^{n} \left[\frac{\lambda_{kj}^{a_{i(j+1)k}-a_{ijk}-1} e^{-\lambda_{kj}}}{(a_{i(j+1)k}-a_{ijk}-1)!} \right]^{\mathbb{I}(w_i=k)}}{\prod_{i=1}^{n} \left[\sum_{x=0}^{L_i-J_k+j-a_{ijk}} \frac{\lambda_{kj}^{x} e^{-\lambda_{kj}}}{x!} \right]^{\mathbb{I}(w_i=k)}} \times \frac{\nu_{kj}^{\beta_{kj}}}{\Gamma(\beta_{kj})} \lambda_{kj}^{\beta_{kj}-1} e^{-\nu_{kj}\lambda_{kj}}$$

$$\propto \frac{\lambda_{kj}^{\sum_{i=1}^{n}(a_{i(j+1)k}-a_{ijk}-1)\mathbb{I}(w_i=k)+\beta_{kj}-1} \cdot e^{-(\sum_{i=1}^{n}\mathbb{I}(w_i=k)+\nu_{kj})\lambda_{kj}}}{\prod_{i=1}^{n} \left(\sum_{x=0}^{L_i-J_k+j-a_{ijk}} \frac{\lambda_{kj}^{x} e^{-\lambda_{kj}}}{x!} \right)^{\mathbb{I}(w_i=k)}}.$$

Since this posterior distribution is non-standard, we use a random walk MH algorithm to obtain posterior samples. Specifically, we apply a normal proposal distribution on the logarithmic scale of λ_{kj}, given that $\lambda_{kj} > 0$:

$$\log \lambda_{kj}^{*} = \log \lambda_{kj} + \epsilon, \quad \epsilon \sim \mathcal{N}(0,\ 0.1^2).$$

We then compute the corresponding acceptance probability to decide whether to accept the proposal λ_{kj}^{*}.

2. Theoretical complexity analysis

Each iteration of the MCMC algorithm updates the latent variables and model parameters, including motif positions $\mathscr{A}$, unknown motif labels $\boldsymbol{W}_U$, Poisson gap parameters Λ, motif distributions ϑ, and the background distribution $\boldsymbol{\theta}_0$. We analyze the per-iteration computational complexity under the assumption of n sequences, K motifs, motif length J, and alphabet size p.

Updating $\mathscr{A}$ involves sampling the motif positions for sequences that are currently assigned to one of the K motifs. For a given sequence, only the row corresponding to its motif label w_i is updated in the corresponding matrix A_{w_i}. Assuming motif labels are approximately uniformly distributed across the K motifs, each matrix A_k is expected to be updated for about n/K sequences. Therefore, the total number of rows updated across all $\mathscr{A}$ matrices is $K \cdot (n/K) = n$. Each updated row contains J motif positions, which are sampled sequentially, one at a time. When updating a particular binding position a_{ijk}, the values of other positions in the same row are held fixed, and the candidate values for a_{ijk} are constrained to lie between $a_{i(j-1)k} + 1$ and $a_{i(j+1)k} - 1$, ensuring valid motif ordering and sequence bounds. Assuming motif positions are approximately uniformly

distributed across the sequence, the expected number of candidates for each position is $O(L/J)$. As there are J positions per sequence, the total cost per sequence is $O(J \cdot L/J) = O(L)$. Aggregated over n sequences, this yields an overall complexity of $O(nL)$ for updating $\mathscr{A}$ per iteration.

Updating $\boldsymbol{W}_U$ requires computing posterior probabilities over $K+1$ possible classes for each sequence, resulting in $O(nK)$. The update of Poisson gap parameters Λ involves $K(J-1)$ Metropolis-Hastings steps, yielding $O(KJ)$. For the motif matrices ϑ, each motif has J categorical distributions over p symbols, leading to $O(KJP)$ complexity. The background distribution $\boldsymbol{\theta}_0$ is updated using counts over all non-motif positions and has complexity $O(P)$.

Combining these steps, the total per-iteration complexity is $O(nL + nK + KJ + KJP + P)$. In typical biological applications, n and L are much larger than K, J, and P. Therefore, the overall complexity simplifies to $O(nL)$.

We also compare this with a gap-free variant, where motifs are assumed to be contiguous. In that case, only the first position of each motif needs to be updated per sequence, resulting in $O(n(L - J + 1)) = O(nL)$ complexity. Thus, the asymptotic complexity remains the same in both cases. However, we emphasize that the sampling behaviors are different. In the gap-aware model, the full space of valid motif positions is not exhaustively enumerated. Instead, positions are updated sequentially under local constraints, which may increase the risk of the sampler becoming trapped in local modes. We mitigate this issue by incorporating a MH shift move that jointly proposes coordinated updates to the motif positions, motif distributions, and gap parameters. This move improves posterior exploration without increasing the overall computational complexity. Therefore, although the gap-aware formulation introduces additional latent variables, the theoretical runtime remains efficient and comparable to the gap-free case.

3. Shift move to jump out of the local mode based on MH algorithm

Here we propose a shift move to jump out of this kind of local mode. Given the strong interdependencies among variables, particularly between the k-th binding locations, the motif matrix $\boldsymbol{\Theta}_k$, and the Poisson gap parameters $\boldsymbol{\lambda}_k$, proposing updates to the binding locations alone often leads to low acceptance rates in the MH procedure. To mitigate this issue, we first shift the current binding locations and also proposes new values for the motif matrix $\boldsymbol{\Theta}_k$ and the gap parameters $\boldsymbol{\lambda}_k$. In each MH proposal, we select a contiguous block of l columns within $\boldsymbol{A}_k$ and

attempt to shift this block by one position, either forward or backward. The shift creates a vacancy at one end of the block, which is filled by proposing a new column of binding locations. Corresponding updates are then applied to the motif matrix $\boldsymbol{\Theta}_k$ and the gap parameters $\boldsymbol{\lambda}_k$, ensuring consistency across all components of the model. The full procedure is detailed below.

Step 1: Proposal for the binding location matrix $\boldsymbol{A}_k^*$

1) Select a block length. A collection of candidate block lengths is considered, where each length l satisfies $l \in \{1, 2, \ldots, J_k - 1\}$. Each candidate represents the size of a contiguous block of columns in $\boldsymbol{A}_k$ that may be shifted.

2) For the sampled block length l, a starting position s is randomly chosen, and the corresponding block $[\boldsymbol{A}_{sk}, \ldots, \boldsymbol{A}_{(s+l-1)k}]$ is selected.

3) For the chosen block, there are two possible shift directions: a forward shift ($\delta = +1$) and a backward shift ($\delta = -1$). One of these directions is selected with equal probability, and the entire block is then shifted by one position in the chosen direction.

4) After the shift, a new column of binding locations is introduced at the vacated position. If the shift is forward, a new column $\boldsymbol{A}_{sk}^*$ is uniformly sampled and inserted at the start of the block. If the shift is backward, a new column $\boldsymbol{A}_{(s+l-1)k}^*$ is sampled and appended at the end. Then the proposal distribution for $\boldsymbol{A}_k^*$ is:

$$p(\boldsymbol{A}_k^* \mid -) \propto \begin{cases} \prod_{i=1}^n \frac{1}{a_{i(s+1)k}^* - a_{i(s-1)k}^* - 1} & \text{if } \delta = +1, \\ \prod_{i=1}^n \frac{1}{a_{i(s+l)k}^* - a_{i(s+l-2)k}^* - 1} & \text{if } \delta = -1. \end{cases}$$

Step 2: Proposal for the motif matrix $\boldsymbol{\Theta}_k^*$ and the gap parameters $\boldsymbol{\lambda}_k^*$

1) The block of motif parameters corresponding to the shifted binding positions, i.e., $[\boldsymbol{\Theta}_{\cdot,sk}, \ldots, \boldsymbol{\Theta}_{\cdot,(s+l-1)k}]$, is shifted in the same direction δ as in Step 1. A new column of motif parameters is then generated for the vacated position. Specifically:

   - If $\delta = +1$, the new motif column is sampled as

$$\boldsymbol{\Theta}_{\cdot,sk}^* \sim \text{Dirichlet}\left(\boldsymbol{H}_{\boldsymbol{A}_{sk}^*} + \boldsymbol{\alpha}_{1k}\right),$$

where $\boldsymbol{H}_{\boldsymbol{A}^*_{sk}} = \sum_{i=1}^n h(r_{i,a^*_{isk}}) \cdot \mathbb{I}(w_i = k)$.

- If $\delta = -1$, the new motif column is sampled as

$$\boldsymbol{\Theta}^*_{\cdot,(s+l-1)k} \sim \text{Dirichlet}\left(\boldsymbol{H}_{\boldsymbol{A}^*_{(s+l-1)k}} + \boldsymbol{\alpha}_{(s+l-1)k}\right),$$

where $\boldsymbol{H}_{\boldsymbol{A}^*_{(s+l-1)k}} = \sum_{i=1}^n h(r_{i,a^*_{i(s+l-1)k}}) \cdot \mathbb{I}(w_i = k)$.

2) For each Poisson gap parameter λ_{kj}, a new value is proposed using a Gaussian random walk in the log space:

$$\log \lambda^*_{kj} = \log \lambda_{kj} + \epsilon, \quad \epsilon \sim \mathcal{N}(0, 0.1^2).$$

Step 3: Acceptance or rejection of the proposal

1) The MH acceptance probability α is computed as follows. If the shift is forward ($\delta = +1$):

$$\min\left\{\frac{\pi(\boldsymbol{A}^*_k, \boldsymbol{\Theta}^*_k, \boldsymbol{\lambda}^*_k \mid -)}{\pi(\boldsymbol{A}_k, \boldsymbol{\Theta}_k, \boldsymbol{\lambda}_k \mid -)} \cdot \frac{p(\boldsymbol{A}_k \mid -) \cdot p(\boldsymbol{\Theta}_{\cdot sk} \mid \boldsymbol{A}_{sk}, -)}{p(\boldsymbol{A}^*_k \mid -) \cdot p(\boldsymbol{\Theta}^*_{\cdot sk} \mid \boldsymbol{A}^*_{sk}, -)}, 1\right\}.$$

If the shift is backward ($\delta = -1$), the acceptance ratio becomes:

$$\min\left\{\frac{\pi(\boldsymbol{A}^*_k, \boldsymbol{\Theta}^*_k, \boldsymbol{\lambda}^*_k \mid -)}{\pi(\boldsymbol{A}_k, \boldsymbol{\Theta}_k, \boldsymbol{\lambda}_k \mid -)} \cdot \frac{p(\boldsymbol{A}_k \mid -)}{p(\boldsymbol{A}^*_k \mid -)} \frac{p(\boldsymbol{\Theta}_{\cdot(s+l-1)k} \mid \boldsymbol{A}_{(s+l-1)k}, -)}{p(\boldsymbol{\Theta}^*_{\cdot(s+l-1)k} \mid \boldsymbol{A}^*_{(s+l-1)k}, -)}, 1\right\}.$$

The joint posterior distribution is given by:

$$\pi(\boldsymbol{A}_k, \boldsymbol{\Theta}_k, \boldsymbol{\lambda}_k \mid -) \propto p(\boldsymbol{R}, \boldsymbol{W}_{U^c} \mid \boldsymbol{W}_U, \mathscr{A}, \vartheta, \boldsymbol{\theta}_0, \Lambda) p(\boldsymbol{A}_k \mid \boldsymbol{\lambda}_k) \cdot p(\boldsymbol{\lambda}_k) \cdot p(\boldsymbol{\Theta}_k).$$

2) A uniform random variable $u \sim \text{Uniform}(0,1)$ is drawn. If $u \leq \alpha$, the proposed values $(\boldsymbol{A}^*_k, \boldsymbol{\Theta}^*_k, \boldsymbol{\lambda}^*_k)$ are accepted as the new state. Otherwise, the current state is retained.

4. Algorithm details

We begin the inference procedure by initializing the parameters $\boldsymbol{W}_U$, $\boldsymbol{A}_k$, $\boldsymbol{\lambda}_k$, $\boldsymbol{\Theta}_k$, and $\boldsymbol{\theta}_0$. Each element of $\boldsymbol{W}_U$ is independently sampled from a Categorical distribution with uniform probability $1/(K+1)$. For each row of $\boldsymbol{A}_k$, the first binding location is drawn from a uniform Categorical distribution. The Poisson gap parameters $\boldsymbol{\lambda}_k$ are then sampled independently from a Gamma

distribution with shape and rate parameters equal to 1, i.e., Gamma$(1, 1)$. The remaining positions in $\boldsymbol{A}_k$ are determined according to a truncated Poisson distribution, Pois$_+$, parameterized by the corresponding entries in $\boldsymbol{\lambda}_k$. Finally, each column of the motif matrix $\boldsymbol{\Theta}_k$, along with the background distribution $\boldsymbol{\theta}_0$, is initialized by sampling from a symmetric Dirichlet distribution, Dirichlet$(\mathbf{1})$.

Following initialization, we iteratively update the parameters $\boldsymbol{W}_U$, $\boldsymbol{A}_k$, $\boldsymbol{\lambda}_k$, $\boldsymbol{\Theta}_k$, and $\boldsymbol{\theta}_0$ in sequence. To escape local modes, a shift move is incorporated every 10 iterations. We adopt the progressive block search strategy as the default for selecting the block length l: during each shift attempt, proposals with $l = 1$ to $l = J_k - 1$ are sequentially evaluated, and the first accepted proposal is used.

After the burn-in period, posterior samples of all model parameters and latent variables are collected. Point estimates are then computed using maximum a posteriori (MAP) estimation, which identifies the values that maximize the joint posterior distribution. The complete procedure is summarized in the GAMMA algorithm (Algorithm 1).

Since the MAP estimate corresponds to a single posterior mode and does not involve averaging over multiple posterior samples or chains, the well-known label switching issue in Bayesian mixture models does not affect our results. For tasks that require posterior averaging or multi-chain analysis, post hoc relabeling techniques could be applied if label switching is observed.

5. Performance for single-motif case

The absolute errors for $\widehat{\boldsymbol{\theta}}_0$ and $\widehat{\boldsymbol{\Theta}}_1$ in the Simulation section are reported in Tables 1.

6. Performance for multiple-motif case

We further evaluate the algorithm's performance in a more complex setting involving three distinct motifs. Specifically, we simulate $n = 200$ sequences of length 10, with the background distribution $\boldsymbol{\theta}_0$ follows $Dirichlet(\mathbf{1})$. The motif lengths for all three motifs are set equal and denoted by J, which is chosen from the set $\{4, 5\}$. Each motif $k = 1, 2, 3$ has its own Dirichlet parameter η_k, with $\boldsymbol{\eta} = (\eta_1, \eta_2, \eta_3)$ set either to $(0.1, 0.1, 0.1)$ or $(0.05, 0.1, 0.2)$, representing different levels of conservation. Motif labels $\boldsymbol{W}$ follow a categorical distribution $Cat(0.3, 0.3, 0.3, 0.1)$, where the first three categories correspond to the three motifs and the fourth represents background (no motif).

Algorithm 1 GAMMA Algorithm

Initialization:

Sample each $w_{u_i} \sim \text{Categorical(Uniform)}$

for each motif k **do**

  Sample first position in each row of $\boldsymbol{A}_k$ from Categorical(Uniform)

  for each i **do**

    Sample $\lambda_{k,i} \sim \text{Gamma}(1, 1)$

    Sample subsequent positions in row i of $\boldsymbol{A}_k$ from $\text{Pois}_+(\lambda_{k,i})$

for each column j of each $\boldsymbol{\Theta}_k$ **do**

  Sample $\boldsymbol{\Theta}_{k,j} \sim \text{Dirichlet}(\mathbf{1})$

Sample background parameter $\boldsymbol{\theta}_0 \sim \text{Dirichlet}(\mathbf{1})$

Posterior Sampling:

for each iteration **do**

  Update $w_{u_i} \sim \text{Cat}(p_{\text{pos}})$

  for each sequence i and motif k **do**

    for $j = 1$ to J_k **do**

      Sample a_{ijk} from $\text{Cat}(\boldsymbol{\pi}_{\text{pos},a_{ijk}})$

  for each position j and component k **do**

    Sample $\boldsymbol{\Theta}_{\cdot jk} \sim \text{Dirichlet}(\boldsymbol{H}_{\boldsymbol{A}_{jk}} + \boldsymbol{\alpha}_{jk})$

  Sample background distribution $\boldsymbol{\theta}_0 \sim \text{Dirichlet}(\boldsymbol{H}_0 + \boldsymbol{\alpha}_0)$

  for each position j and component k **do**

    Propose $\log \lambda^*_{kj}$ and accept with MH probability

  if iteration mod $10 = 0$ **then**

    Perform shift moves

Post-processing:

Discard burn-in samples and compute MAP estimates

Table 1: Result for single-motif case

	$J_1 = 5 \mid \eta = 0.1 \mid S = \{0.5, 1, 1.5\}$						$J_1 = 5 \mid \eta = 0.2 \mid S = \{0.5, 1, 1.5\}$					
		$\lvert\widehat{\boldsymbol{\Theta}}_{j1} - \boldsymbol{\Theta}_{j1}\rvert$						$\lvert\widehat{\boldsymbol{\Theta}}_{j1} - \boldsymbol{\Theta}_{j1}\rvert$				
Letter	$\lvert\widehat{\boldsymbol{\theta}_0} - \boldsymbol{\theta}_0\rvert$	$j = 1$	$j = 2$	$j = 3$	$j = 4$	$j = 5$	$\lvert\widehat{\boldsymbol{\theta}_0} - \boldsymbol{\theta}_0\rvert$	$j = 1$	$j = 2$	$j = 3$	$j = 4$	$j = 5$
A	0.003	0	0.016	0.059	0.009	0.011	0	0.01	0.007	0.021	0	0.01
C	0.008	0.004	0.005	0.033	0.005	0.001	0.004	0.013	0.003	0.011	0.009	0.011
D	0.005	0.01	0.015	0.016	0.002	0.013	0	0.007	0.006	0.009	0.011	0.009
E	0	0.001	0.003	0.007	0.002	0.007	0.01	0.002	0.058	0.003	0.004	0.03
F	0.009	0.01	0.09	0.002	0.012	0.015	0.002	0.076	0.011	0.08	0.001	0.014
G	0.011	0.01	0.008	0.008	0	0.008	0.01	0.116	0.013	0.002	0.026	0.001
H	0.004	0.004	0.011	0.02	0.006	0.001	0.008	0.007	0.006	0.004	0.003	0.005
I	0.008	0.005	0.007	0.002	0	0.001	0.026	0.006	0.009	0.005	0.002	0.052
K	0.001	0.009	0	0.013	0.006	0.011	0.005	0.022	0.003	0.077	0.013	0.002
L	0.011	0	0.001	0.007	0.034	0.019	0	0.007	0.01	0	0.011	0.046
M	0.016	0.011	0.134	0.001	0	0.021	0.009	0.012	0.006	0.011	0.029	0.009
N	0.002	0	0.007	0	0.001	0.012	0.023	0.016	0.036	0.01	0.046	0.024
P	0.005	0.008	0.002	0	0.006	0.016	0.002	0.01	0.047	0.017	0.029	0.021
Q	0.004	0.031	0.004	0.007	0.021	0.086	0.003	0.002	0.003	0.008	0.003	0.002
R	0.008	0	0.088	0.015	0.023	0.01	0.006	0.015	0.012	0.008	0	0
S	0	0.014	0.023	0.038	0.003	0.001	0	0.039	0.033	0.013	0.017	0.046
T	0.008	0.05	0.005	0.012	0.001	0.018	0.009	0.008	0.005	0.008	0.017	0.023
V	0	0.013	0.014	0.036	0.022	0.004	0.001	0.007	0.054	0.003	0.018	0.068
W	0.018	0.002	0.007	0.009	0.006	0.002	0	0.006	0.004	0.01	0.01	0.016
Y	0.001	0.006	0.012	0.002	0.001	0.055	0.033	0.058	0.006	0.013	0.032	0.001
		$J_1 = 4 \mid \eta = 0.1 \mid S = \{0.5, 1, 1.5\}$						$J_1 = 5 \mid \eta = 0.1 \mid S = \{1.5, 2, 2.5\}$				
		$\lvert\widehat{\boldsymbol{\Theta}}_{j1} - \boldsymbol{\Theta}_{j1}\rvert$						$\lvert\widehat{\boldsymbol{\Theta}}_{j1} - \boldsymbol{\Theta}_{j1}\rvert$				
Letter	$\lvert\widehat{\boldsymbol{\theta}_0} - \boldsymbol{\theta}_0\rvert$	$j = 1$	$j = 2$	$j = 3$	$j = 4$		$\lvert\widehat{\boldsymbol{\theta}_0} - \boldsymbol{\theta}_0\rvert$	$j = 1$	$j = 2$	$j = 3$	$j = 4$	$j = 5$
A	0.003	0.002	0.003	0.006	0.033		0	0.01	0.007	0.021	0	0.01
C	0.002	0.001	0.003	0.002	0.006		0.004	0.013	0.003	0.011	0.009	0.011
D	0	0.011	0	0.004	0.037		0	0.007	0.006	0.009	0.011	0.009
E	0.007	0.008	0.004	0.03	0.034		0.01	0.002	0.058	0.003	0.004	0.03
F	0.019	0.008	0.003	0.007	0.035		0.002	0.076	0.011	0.08	0.001	0.014
G	0.006	0.003	0.006	0.023	0.052		0.01	0.116	0.013	0.002	0.026	0.001
H	0.005	0.013	0.017	0.001	0.016		0.008	0.007	0.006	0.004	0.003	0.005
I	0.003	0.003	0.014	0.023	0.001		0.026	0.006	0.009	0.005	0.002	0.052
K	0.02	0.005	0.001	0.019	0.001		0.005	0.022	0.003	0.077	0.013	0.002
L	0.008	0.006	0	0.009	0.005		0	0.007	0.01	0	0.011	0.046
M	0.017	0.005	0.001	0.002	0.017		0.009	0.012	0.006	0.011	0.029	0.009
N	0.002	0.006	0.004	0.012	0.02		0.023	0.016	0.036	0.01	0.046	0.024
P	0	0.006	0.042	0.004	0.01		0.002	0.01	0.047	0.017	0.029	0.021
Q	0.005	0.086	0.001	0.001	0.003		0.003	0.002	0.003	0.008	0.003	0.002
R	0.002	0	0.01	0.018	0.002		0.006	0.015	0.012	0.008	0	0
S	0.011	0.013	0.001	0.033	0.002		0	0.039	0.033	0.013	0.017	0.046
T	0.001	0.005	0.005	0.016	0.002		0.009	0.008	0.005	0.008	0.017	0.023
V	0.01	0.012	0.005	0.041	0.075		0.001	0.007	0.054	0.003	0.018	0.068
W	0.002	0.005	0	0.026	0.006		0	0.006	0.004	0.01	0.01	0.016
Y	0.003	0.013	0.003	0.02	0.073		0.033	0.058	0.006	0.013	0.032	0.001

The Poisson parameters λ_{kj}, which control the spacing between binding locations, are drawn from the set $S = \{0.5, 1.0, 1.5\}$. Binding site locations are generated in the same manner as in the single-motif setting. To simulate incomplete annotations as encountered in real biological datasets, we introduce label masking by randomly setting 20% of the entries in $\boldsymbol{W}$ to 'NA'. We also employ a non-informative prior in this case. The algorithm is executed for 1,000 iterations, with the initial 500 iterations discarded as burn-in. All parameters are initialized by sampling from their respective prior distributions.

The algorithm's performance is evaluated using the same procedure as in the single-motif setting. Results are summarized in Figure 1, where each experimental condition is defined by a pair $(J, \boldsymbol{\eta})$, with J denoting the common motif length across the three motifs, and $\boldsymbol{\eta}$ representing the Dirichlet concentration parameters for $\boldsymbol{\Theta}_{j1}$, $\boldsymbol{\Theta}_{j2}$, and $\boldsymbol{\Theta}_{j3}$. As shown in the figure, the algorithm demonstrates consistently strong performance across different combinations of motif lengths and conservation levels. These results highlight the flexibility and robustness of the algorithm in modeling complex biological scenarios involving multiple motifs.

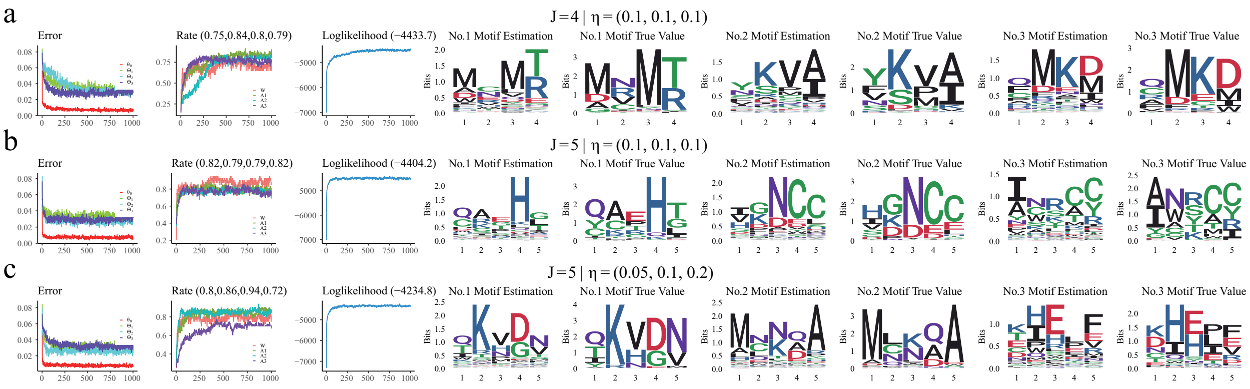

Figure 1: **Inference performance under multiple-motif simulation scenario.** The first column presents trace plots of errors, where red, green, blue and purple curves correspond to $\boldsymbol{\theta}_0$, $\boldsymbol{\Theta}_1$, $\boldsymbol{\Theta}_2$, and $\boldsymbol{\Theta}_3$, respectively. The second column shows accuracy trace plots for $\boldsymbol{W}$ (red), $\boldsymbol{A}_1$ (green), $\boldsymbol{A}_2$ (blue) and $\boldsymbol{A}_3$ (purple). The third column illustrates the log-likelihood trace plots. The fourth through ninth columns display sequence logos: estimated $\boldsymbol{\Theta}_1$, true $\boldsymbol{\Theta}_1$, estimated $\boldsymbol{\Theta}_2$, true $\boldsymbol{\Theta}_2$, estimated $\boldsymbol{\Theta}_3$ and true $\boldsymbol{\Theta}_3$ respectively.

The absolute errors for $\widehat{\boldsymbol{\theta}}_0$, $\widehat{\boldsymbol{\Theta}}_1$, $\widehat{\boldsymbol{\Theta}}_2$ and $\widehat{\boldsymbol{\Theta}}_3$ in the Simulation section are reported in Tables 2.

Overall, simulation studies under both single- and multiple-motif settings demonstrate that GAMMA is robust and accurate. In single-motif cases, it reliably identifies motif locations and patterns across different lengths, conservation levels, and spacings, even without partial labels. In the more complex multiple-motif setting, where each sequence contains at most one of several motif

Table 2: Result for multiple-motif case

$J = 4 \mid \eta = (0.1, 0.1, 0.1)$

		$\lvert\widehat{\boldsymbol{\Theta}}_{j1} - \boldsymbol{\Theta}_{j1}\rvert$				$\lvert\widehat{\boldsymbol{\Theta}}_{j2} - \boldsymbol{\Theta}_{j2}\rvert$				$\lvert\widehat{\boldsymbol{\Theta}}_{j3} - \boldsymbol{\Theta}_{j3}\rvert$			
Letter	$\lvert\widehat{\boldsymbol{\theta}}_0 - \boldsymbol{\theta}_0\rvert$	$j=1$	$j=2$	$j=3$	$j=4$	$j=1$	$j=2$	$j=3$	$j=4$	$j=1$	$j=2$	$j=3$	$j=4$
A	0.002	0.009	0.009	0.005	0.014	0.016	0.046	0.002	0.035	0.063	0.006	0.043	0.003
C	0.01	0.024	0.04	0.026	0.014	0.029	0	0.01	0.001	0.047	0.019	0.048	0.006
D	0	0.041	0.017	0.028	0.016	0.027	0.021	0.003	0.02	0.008	0.009	0.002	0.201
E	0	0.002	0.091	0.025	0.075	0.001	0.014	0.019	0.006	0.027	0.002	0.051	0.004
F	0.002	0.038	0.005	0.032	0.002	0.057	0.001	0.007	0	0.071	0.007	0.038	0.008
G	0.002	0.064	0.002	0.016	0.032	0.027	0.019	0.013	0.009	0.012	0	0.007	0.008
H	0.007	0.011	0.037	0.003	0.008	0.001	0.013	0.013	0.02	0.06	0.017	0.024	0.021
I	0	0.038	0.016	0.015	0.013	0.042	0.035	0.005	0.081	0.004	0.006	0.005	0.014
K	0.001	0.028	0	0.001	0.007	0.009	0.171	0.035	0.062	0.007	0.023	0.126	0.015
L	0.011	0.01	0.024	0.047	0.005	0.018	0.017	0.011	0.008	0.016	0.011	0.006	0.031
M	0.028	0.194	0.04	0.335	0.002	0.001	0	0.109	0.015	0.005	0.181	0.004	0.084
N	0.003	0.015	0.084	0.004	0.02	0.041	0.033	0.004	0.016	0.037	0.018	0.054	0.032
P	0.01	0.039	0.012	0.005	0.001	0.024	0.028	0.147	0.045	0.04	0.011	0.009	0.001
Q	0.011	0.003	0.01	0.056	0	0.042	0.019	0.041	0.016	0.034	0.034	0.026	0.024
R	0.002	0.005	0.161	0.016	0.05	0.036	0.059	0.002	0.001	0.084	0.017	0.025	0.003
S	0.003	0.002	0.001	0.005	0.006	0.001	0.079	0.06	0.004	0.002	0.014	0.015	0.003
T	0.004	0.006	0.017	0.008	0.128	0.006	0.004	0.006	0.002	0.011	0.016	0.006	0.003
V	0.007	0.002	0.12	0.062	0.002	0.072	0.004	0.065	0	0.068	0.004	0.001	0.007
W	0.011	0.094	0.047	0.012	0.007	0.014	0.002	0.001	0.004	0.002	0.006	0.006	0.047
Y	0.001	0.005	0.029	0.008	0.002	0.129	0.017	0.024	0.01	0.014	0.03	0.021	0

$J = 5 \mid \eta = (0.1, 0.1, 0.1)$

		$\lvert\widehat{\boldsymbol{\Theta}}_{j1} - \boldsymbol{\Theta}_{j1}\rvert$					$\lvert\widehat{\boldsymbol{\Theta}}_{j2} - \boldsymbol{\Theta}_{j2}\rvert$					$\lvert\widehat{\boldsymbol{\Theta}}_{j3} - \boldsymbol{\Theta}_{j3}\rvert$				
Letter	$\lvert\widehat{\boldsymbol{\theta}}_0 - \boldsymbol{\theta}_0\rvert$	$j=1$	$j=2$	$j=3$	$j=4$	$j=5$	$j=1$	$j=2$	$j=3$	$j=4$	$j=5$	$j=1$	$j=2$	$j=3$	$j=4$	$j=5$
A	0	0.012	0.193	0.003	0.005	0.004	0.008	0.007	0.032	0.002	0.009	0.221	0.037	0.019	0.001	0.017
C	0.015	0.045	0.123	0.059	0.026	0.003	0.043	0.006	0.005	0.088	0.065	0.019	0.083	0.043	0.165	0.023
D	0.009	0.048	0.015	0.008	0.024	0.015	0.008	0.096	0.052	0.027	0.003	0.006	0.01	0.033	0.014	0.031
E	0.013	0.001	0.028	0.12	0.005	0.014	0.005	0	0.024	0.07	0.139	0.036	0.005	0.008	0.015	0.007
F	0.013	0.035	0.001	0.024	0.012	0.004	0.008	0.049	0.022	0.007	0.008	0.001	0.01	0.004	0.04	0.001
G	0.01	0.002	0.029	0.04	0.009	0.006	0.052	0.147	0.004	0.021	0.016	0.07	0.044	0.001	0.006	0.013
H	0.004	0.045	0.011	0.042	0.168	0.036	0.186	0	0.004	0.006	0.005	0	0.033	0.01	0.026	0.003
I	0.005	0.025	0.065	0.002	0.003	0.038	0.064	0.011	0.007	0.006	0.011	0.03	0.002	0.001	0.015	0.121
K	0.002	0.01	0.023	0.003	0.01	0.02	0.004	0.008	0.012	0.003	0.01	0.009	0.081	0.033	0.012	0.013
L	0.02	0.023	0.036	0.057	0.045	0.036	0.018	0.046	0.001	0.001	0.02	0.001	0.018	0.07	0.029	0.026
M	0.002	0.016	0.007	0.016	0.001	0.001	0.004	0.021	0.005	0.022	0.026	0.008	0.004	0.017	0.028	0.012
N	0.007	0.017	0.004	0.001	0.015	0.008	0.026	0.005	0.113	0.008	0.023	0.06	0.185	0.011	0.002	0.005
P	0.01	0.069	0	0.05	0.014	0.014	0.004	0.015	0.028	0.004	0.027	0.052	0.002	0.002	0.004	0.001
Q	0.001	0.151	0.008	0	0.034	0.04	0.006	0.004	0.005	0.006	0.022	0.001	0.016	0.029	0.005	0.003
R	0.008	0.007	0.099	0.093	0.003	0.045	0.006	0	0.011	0.002	0.001	0.019	0.015	0.018	0.059	0.049
S	0.009	0.005	0.069	0.002	0.008	0.01	0.038	0.035	0.012	0.007	0.06	0.003	0.099	0.073	0.012	0.016
T	0.012	0.025	0.035	0.005	0.008	0.242	0.015	0.012	0.01	0.009	0.018	0.009	0.012	0.076	0.03	0.017
V	0.005	0.004	0.006	0.012	0.018	0.047	0.069	0.002	0.014	0.039	0.015	0.047	0.033	0.001	0.003	0.002
W	0.018	0.038	0.015	0.015	0.007	0.009	0.001	0.004	0.005	0.03	0.01	0.021	0.088	0.013	0.009	0.018
Y	0.015	0.168	0.009	0.037	0.007	0.001	0.018	0.018	0.01	0.027	0.01	0.016	0.017	0.098	0.012	0.004

$J = 5 \mid \eta = (0.05, 0.1, 0.2)$

		$\lvert\widehat{\boldsymbol{\Theta}}_{j1} - \boldsymbol{\Theta}_{j1}\rvert$					$\lvert\widehat{\boldsymbol{\Theta}}_{j2} - \boldsymbol{\Theta}_{j2}\rvert$					$\lvert\widehat{\boldsymbol{\Theta}}_{j3} - \boldsymbol{\Theta}_{j3}\rvert$				
Letter	$\lvert\widehat{\boldsymbol{\theta}}_0 - \boldsymbol{\theta}_0\rvert$	$j=1$	$j=2$	$j=3$	$j=4$	$j=5$	$j=1$	$j=2$	$j=3$	$j=4$	$j=5$	$j=1$	$j=2$	$j=3$	$j=4$	$j=5$
A	0.002	0.019	0.007	0.005	0.012	0.02	0.035	0.02	0.023	0.093	0.187	0.003	0	0.02	0.008	0.005
C	0.007	0.046	0.005	0.033	0.026	0.002	0.01	0.017	0	0.026	0.013	0.01	0.02	0.02	0.017	0.009
D	0.013	0.004	0.005	0.02	0.127	0.01	0.021	0.002	0.003	0.069	0.004	0.134	0.011	0.001	0.061	0.078
E	0.002	0.004	0.005	0	0.013	0.006	0.003	0.053	0.006	0.054	0.004	0.092	0.03	0.061	0.027	0.068
F	0.01	0.004	0.01	0.001	0.01	0.011	0.01	0.016	0.011	0.01	0.005	0.002	0.017	0.022	0.084	0.061
G	0.013	0.017	0.003	0.006	0.029	0.023	0.009	0.011	0.018	0.02	0.005	0.005	0.028	0.025	0.041	0.013
H	0.002	0.001	0.005	0.112	0.002	0.027	0.049	0.004	0.008	0	0.013	0.003	0.27	0.242	0.009	0.013
I	0.001	0.024	0.011	0	0.009	0.004	0.013	0.004	0.017	0.006	0.001	0.017	0.061	0.009	0.115	0.046
K	0.012	0.052	0.232	0.004	0	0.023	0.006	0.001	0.047	0.002	0	0.074	0.005	0.003	0.021	0
L	0.003	0.021	0.003	0.054	0.015	0.043	0.073	0.174	0.015	0.022	0.002	0.021	0.002	0.036	0.05	0.025
M	0.013	0.001	0.019	0.068	0.009	0.009	0.228	0.002	0.017	0.024	0.009	0.022	0.024	0.015	0.005	0.049
N	0.005	0.025	0.012	0.035	0.003	0.332	0.01	0.023	0.014	0.024	0.007	0.064	0.001	0.022	0.007	0.03
P	0.002	0.015	0.005	0.07	0.007	0.006	0.012	0.008	0.005	0.006	0.039	0.018	0.091	0.015	0.07	0.018
Q	0.009	0.109	0.013	0.014	0.012	0.003	0	0.005	0.007	0.183	0.013	0.029	0	0.011	0.021	0.017
R	0.006	0.003	0.009	0.008	0.012	0.02	0.002	0.021	0.008	0.006	0.05	0.005	0.014	0.06	0.017	0.017
S	0.002	0.028	0.036	0.008	0.005	0.067	0.01	0.01	0.003	0.025	0.008	0.021	0.022	0.04	0.009	0.01
T	0.018	0.054	0.053	0.026	0.002	0.067	0.004	0.004	0.061	0.012	0.018	0.081	0.007	0.001	0.027	0.002
V	0.007	0.025	0.015	0.244	0.021	0.029	0.002	0	0.005	0.003	0.006	0.006	0.018	0.021	0.016	0.06
W	0.002	0.064	0.008	0.005	0.016	0.021	0.011	0.018	0.009	0.015	0.022	0.003	0.053	0.019	0.023	0.01
Y	0.001	0.092	0.008	0.015	0.014	0.011	0.019	0.001	0.03	0.011	0.027	0.017	0.001	0.009	0.014	0.001

types, GAMMA accurately identifies the motif type and reconstructs its pattern. These results confirm the model's strong capability in motif classification and localization.

7. Demonstration of shift move escaping a local mode

To visualize the effect of the MH-based shift move and its role in escaping local modes, we conducted a targeted simulation experiment following the structure of the toy example presented in Figure 3 of the main text.

We generated synthetic data consisting of sequences of length 9. The true motif binding locations were fixed at positions 1, 4, 6, 8, and 9. The motif matrix was initialized in a misaligned configuration, with binding positions at 1, 2, 4, 6, and 9. This setting creates a local mode that overlaps partially with the true motif but is offset by one position for the central columns.

We used non-informative priors for all model parameters and ran the MCMC sampler as described previously. During sampling, we observed that the sampler initially remained trapped in a local mode, with the estimated motif matrix misaligned. At a certain iteration, a shift move was accepted that updated a block of columns and realigned the estimated motif with the true motif structure.

Figure 2 illustrates this process. The first panel shows the estimated motif before the shift move, which is clearly misaligned. Specifically, columns 3 and 4 in the estimated motif correspond to columns 2 and 3 in the ground truth. The second panel shows the motif after the shift move, which aligns closely with the true motif shown in the third panel.

This example provides concrete visual evidence that the shift move enables the sampler to escape from a local posterior mode and realign the motif correctly. Without such a move, the sampler would have remained stuck in a suboptimal configuration due to the discreteness of the binding location space.

8. Impact of block length on MCMC Performance

To examine the influence of block length on the efficiency and convergence of the MCMC algorithm, we designed an additional simulation experiment under a simplified single-motif setting. In this experiment, we simulate $n = 200$ biological sequences, each of length 9. The background distribution parameters $\boldsymbol{\theta}_0$ are drawn from a symmetric Dirichlet prior Dirichlet$(\mathbf{1})$, and the motif

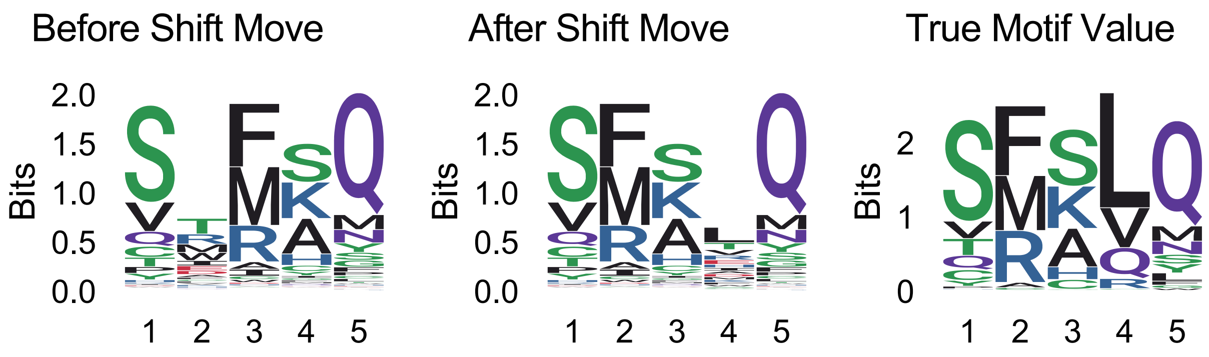

Figure 2: Illustration of the shift move escaping a local mode. From left to right: the motif estimated before the shift move, the motif after applying the shift move, and the ground-truth motif. In the initial local mode, the motif is misaligned, particularly in the central positions. After the shift move, the motif aligns closely with the true motif, demonstrating the effectiveness of this move in improving posterior exploration.

length is fixed at 5. For each motif position $j = 1, \ldots, 4$, the corresponding column of the motif matrix $\boldsymbol{\Theta}_{j1}$ is generated from a Dirichlet distribution with concentration parameter 0.1. The motif presence indicator w_i for each sequence is sampled from a categorical distribution $\text{Cat}(0.8, 0.2)$, where $w_i = 1$ indicates the presence of a motif and $w_i = 2$ its absence. The MCMC sampler was implemented using uniform (non-informative) priors for all model parameters and latent variables, including $\boldsymbol{\theta}_0$, $\boldsymbol{\Theta}_{j1}$, and W. Specifically, all admissible values were assigned equal prior probability, consistent with the setup described in the main text. The sampler was run for 1000 iterations, with the first 600 iterations discarded as burn-in.

We tested five configurations. The first configuration, referred to as the progressive block search, sequentially attempts shift proposals with block lengths $l = 1$ through 4 in each iteration. The algorithm begins with $l = 1$ and proceeds to the next value only if the current proposal is rejected, stopping either upon acceptance or after all four values have been attempted without success. The maximum allowable block length is constrained to be the motif length minus one, which is 4 in this case. The remaining four configurations use a fixed block length of $l = 1$, 2, 3, or 4, respectively, with only a single shift proposal evaluated per iteration. In this context, the block length l determines the number of consecutive motif positions (i.e., columns) that are jointly shifted in a proposal. Smaller values of l lead to more localized updates, while larger values enable more global shifts across the motif.

Figure 3 shows the log-likelihood trajectories under these five settings. The first panel corre-

sponds to the progressive block search strategy, while the remaining panels illustrate the results for fixed block lengths of 1 through 4 (from left to right). The vertical dashed line marks the burn-in threshold at iteration 600. Sampling efficiencies for $\boldsymbol{\theta}_0$ and $\boldsymbol{\Theta}_1$, computed after burn-in, are annotated in the top-left corner of each panel. Here, sampling efficiency is defined as the ratio of effective sample size (ESS) to the number of post-burn-in samples, i.e., ESS$/n$. The ESS is estimated using the standard autocorrelation-based method, where higher autocorrelation indicates lower effective sample size and hence reduced efficiency.

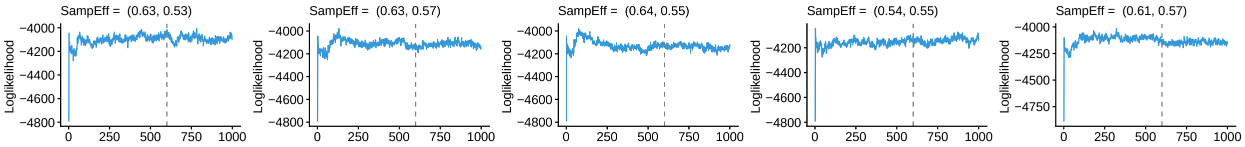

Figure 3: Log-likelihood trajectories under different block length strategies. The first panel corresponds to the progressive block search, which attempts shift proposals sequentially with block lengths $l = 1$ to 4. The remaining panels show results for fixed block lengths of $l = 1$, 2, 3, and 4, respectively. The vertical dashed line marks burn-in at iteration 600. Sample efficiencies for $\boldsymbol{\theta}_0$ and $\boldsymbol{\Theta}_1$ after burn-in are shown in the top-left corner of each panel.

The results in Figure 3 indicate that all five settings produce comparable performance in terms of convergence and sampling efficiency. While there are minor differences in the smoothness of log-likelihood trajectories and efficiency metrics, no single block length consistently outperforms the others. Based on these observations, we adopt the progressive block search as the default strategy. It provides a balance between local and global proposals and eliminates the need for manual tuning of the block length parameter.

9. Effect of shift move frequency

To evaluate the effect of different shift move frequencies in the MH algorithm, we reused the same simulation setup as in the block length experiment. The only difference is that the block length was fixed using the progressive block search strategy, which sequentially attempts shift proposals with $l = 1$ to 4 in each MH step. The shift move frequency, defined as the interval at which the MH shift move is performed, was varied across runs.

Specifically, we compared four frequency settings: performing the shift move every 1, 2, 5, or 10 MCMC iterations. All other parameters, including priors, initializations, and the total number of MCMC iterations (1000 with 600 burn-in), were kept unchanged.

The results are summarized in Table 3. We report the maximum log-likelihood achieved, the mean absolute errors of the estimated background parameter $\boldsymbol{\theta}_0$ and motif matrix $\boldsymbol{\Theta}_1$, as well as the average computation time per replication.

Table 3: Performance metrics under different shift move frequencies

	**Freq = 1**	**Freq = 2**	**Freq = 5**	**Freq = 10**
Maximum Log-Likelihood	-4015.78	-4014.16	-4011.15	-4006.32
Mean Absolute Error of $\boldsymbol{\theta}_0$	0.017	0.020	0.022	0.018
Mean Absolute Error of $\boldsymbol{\Theta}_1$	0.053	0.041	0.042	0.041
Time per Replication (seconds)	0.749	0.516	0.352	0.320

All settings yield comparable estimation accuracy, with only minor differences across shift move frequencies. Notably, performing the shift move less frequently (e.g., every 10 iterations) maintains estimation quality while reducing computation time. The mean absolute error of $\boldsymbol{\Theta}_1$ remains stable (around 0.041-0.042), while the computation time per replication decreases from 0.749 seconds (every iteration) to 0.320 seconds (every 10 iterations). Based on these results, we selected a shift move frequency of every 10 iterations as a balanced default that offers good computational efficiency without compromising estimation accuracy.

10. Impact of parameter correlation between motifs

Our model assumes that parameters across different motifs are conditionally independent given the data and latent variables. In particular, the motif probabilities $\boldsymbol{\Theta}_k$ and the spacing parameters $\boldsymbol{\lambda}_k$ are modeled independently across motifs. To assess the validity and practical implications of this assumption, we conducted a simulation study designed to evaluate potential interactions between parameters in a multi-motif setting.

We simulated datasets consisting of $n = 200$ peptide sequences of length 10. The background amino acid distribution was drawn from a symmetric Dirichlet prior: $\boldsymbol{\theta}_0 \sim \text{Dirichlet}(\mathbf{1})$. Two motifs were included in each dataset, each with a fixed length $J = 5$. For the first motif, each column of the motif matrix was generated independently from a Dirichlet distribution with strong conservation: $\boldsymbol{\Theta}_{1,j} \sim \text{Dirichlet}(0.1)$. To control the degree of similarity between the two motifs, we constructed the second motif matrix $\boldsymbol{\Theta}_2$ as a combination of $\boldsymbol{\Theta}_1$ and a randomly drawn independent motif $\boldsymbol{\Theta}_{\text{raw}}$, where each column $\boldsymbol{\Theta}_{\text{raw},j} \sim \text{Dirichlet}(0.1)$. Specifically,

$$\boldsymbol{\Theta}_{2,j} = \rho\boldsymbol{\Theta}_{1,j} + (1-\rho)\boldsymbol{\Theta}_{\text{raw},j}, \quad j = 1, \ldots, J,$$

where $\rho \in [0, 0.3]$ takes 15 equally spaced values. Smaller ρ values correspond to more distinct motifs, while larger values reflect increasing similarity between $\boldsymbol{\Theta}_1$ and $\boldsymbol{\Theta}_2$.

To assess the effect of spacing differences, we considered three spacing structures. In the homogeneous case, both motifs share identical spacing parameters, with $\boldsymbol{\lambda}_1 = \boldsymbol{\lambda}_2 = (0.5, 0.5, 0.5, 0.5)$. In the partially homogeneous case, we set $\boldsymbol{\lambda}_1 = (0.5, 0.5, 0.5, 0.5)$ and $\boldsymbol{\lambda}_2 = (0.5, 0.5, 1.5, 1.5)$, introducing moderate differences in spacing. In the heterogeneous case, we specified $\boldsymbol{\lambda}_1 = (0.5, 0.5, 0.5, 0.5)$ and $\boldsymbol{\lambda}_2 = (1.5, 1.5, 1.5, 1.5)$, representing strong divergence in spacing patterns between the two motifs.

Motif binding positions were generated recursively using truncated Poisson distributions conditioned on the previous site, following the procedure in the main text. Motif identity labels $\boldsymbol{W} \in \{1, 2, 3\}$ were drawn from $\text{Cat}(0.4, 0.4, 0.2)$, where class 3 represents background (no motif). We randomly masked 20% of the $\boldsymbol{W}$ labels to mimic partially labeled data. Each configuration was repeated over 10 independent simulation replicates with different random seeds. The algorithm was run for 500 MCMC iterations, with the first 200 discarded as burn-in.

To evaluate model performance, we computed the average accuracy in recovering motif labels as a function of the KL divergence between the two motifs, averaged over motif positions:

$$\text{KL}(\boldsymbol{\Theta}_1 \| \boldsymbol{\Theta}_2) = \frac{1}{J}\sum_{j=1}^{J} \text{KL}(\boldsymbol{\Theta}_{1,j} \| \boldsymbol{\Theta}_{2,j}).$$

Figure 4 presents the regression analysis of classification accuracy as a function of KL divergence under three spacing scenarios: homogeneous, partially homogeneous, and heterogeneous. Across all conditions, there is a strong and consistent positive relationship between motif distinctiveness, measured by KL divergence, and label recovery accuracy.

In the heterogeneous setting, where spacing patterns differ substantially, classification accuracy is higher at lower KL divergence compared to the other settings. This suggests that even when motifs are relatively similar, variation in spacing can still contribute to distinguishing between them. The regression slope is steepest in the homogeneous setting, where spacing patterns are identical across motifs. This indicates that when spacing does not provide additional distinguishing

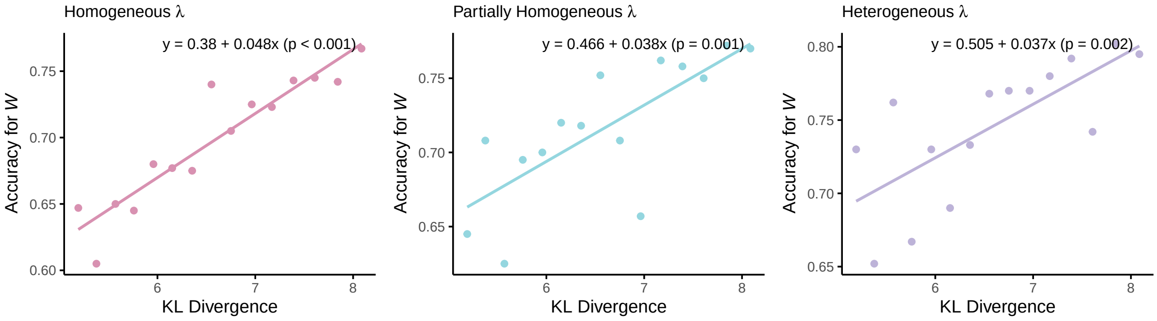

Figure 4: Regression between KL divergence and motif label accuracy under three settings for λ. Each subplot corresponds to a different condition: Homogeneous, Partially Homogeneous, and Heterogeneous. All accuracy values are averaged over 10 random seeds.

information, the model becomes more sensitive to differences in motif content. Summary statistics, including the means and standard deviations of KL divergence and accuracy across different ρ values, are provided in Table 4.

	ρ	0.000	0.021	0.043	0.064	0.086	0.107	0.129	0.150	0.171	0.193	0.214	0.236	0.257	0.279	0.300
	KL Mean	8.086	7.843	7.608	7.391	7.170	6.964	6.752	6.552	6.355	6.151	5.959	5.759	5.571	5.376	5.191
	KL Std	1.357	1.324	1.290	1.259	1.227	1.196	1.165	1.134	1.104	1.073	1.043	1.012	0.982	0.951	0.922
Homogeneous	Accuracy Mean	0.767	0.742	0.745	0.743	0.723	0.725	0.705	0.740	0.675	0.677	0.680	0.645	0.650	0.605	0.647
	Accuracy Std	0.076	0.094	0.083	0.071	0.092	0.082	0.048	0.113	0.100	0.096	0.069	0.071	0.096	0.055	0.079
Partially Homogeneous	Accuracy Mean	0.770	0.772	0.750	0.758	0.762	0.657	0.708	0.752	0.718	0.720	0.700	0.695	0.625	0.708	0.645
	Accuracy Std	0.069	0.098	0.077	0.078	0.076	0.073	0.073	0.082	0.079	0.076	0.073	0.077	0.088	0.095	0.081
Heterogeneous	Accuracy Mean	0.795	0.802	0.742	0.792	0.780	0.770	0.770	0.768	0.733	0.690	0.730	0.667	0.762	0.652	0.730
	Accuracy Std	0.067	0.118	0.059	0.076	0.050	0.071	0.083	0.058	0.062	0.090	0.057	0.097	0.070	0.095	0.125

Table 4: KL divergence and classification accuracy (mean and standard deviation) across 15 values of ρ.

11. Parallelization and computational efficiency

The computational cost of the proposed Bayesian motif model increases with both the number of sequences (n) and the sequence length (L), which may limit its scalability in large-scale applications. To improve computational efficiency, we developed a parallel implementation of the algorithm by utilizing the conditional independence structure of the latent variable updates across sequences.

In particular, the sampling of the latent variables W and A, which represent motif label and motif position respectively, is conditionally independent across sequences given the current model parameters. This structure makes these steps inherently parallelizable. We implemented the parallel version using the `parLapply` function in R and specified the use of 5 CPU cores for parallel

execution. This setup allows the computations to be distributed across the available cores, reducing the overall runtime per iteration.

To evaluate the efficiency gain, we conducted a series of simulation studies under varying numbers of sequences ($n \in \{500, 1000, 2500, 5000, 10000, 20000, 40000\}$) and sequence lengths ($L \in \{10, 15, 30\}$). In each setting, we generated synthetic datasets and measured the average wall-clock time per MCMC iteration over ten repetitions. Both serial and parallel implementations were tested under identical conditions.

The synthetic data were generated based on a predefined motif structure. Specifically, we used the standard 20-letter amino acid alphabet and fixed the motif length to 8. Each sequence was randomly assigned a motif label $W_i \in \{1, 2\}$, indicating whether the motif is present. The presence probability was set to 0.8, and 20% of the labels were randomly masked to simulate missing data. For sequences with a motif, the motif positions a_{ij1} was generated from a truncated Poisson distribution conditional on $a_{i(j-1)1}$, ensuring all motif elements reside within the valid sequence range. The motif regions were drawn from position-specific multinomial distributions sampled from Dirichlet priors with concentration $\eta = 0.1$.

Figure 5 presents the time saved per iteration achieved by the parallel implementation, while Figure 6 compares the absolute execution time between the serial and parallel versions across different sequence lengths and dataset sizes. Detailed timing results are summarized in Table 5. As shown in Figure 5, the benefit of parallelization becomes increasingly evident as the dataset size grows. For example, in Figure 6, when the number of sequences reaches 40,000 and the sequence length is 30, the average iteration time is reduced from 85.26 seconds in the serial implementation to 49.43 seconds in the parallel version, resulting in a time saving of over 35 seconds per iteration.

In contrast, for smaller datasets, the parallel implementation introduces a slight computational overhead. When the number of sequences is limited, such as $n = 500$, the average iteration time in the parallel version can exceed that of the serial one. For instance, with $n = 500$ and $L = 10$, the parallel implementation takes 2.12 seconds per iteration, whereas the serial version requires only 0.68 seconds. This reversal is primarily due to the overhead associated with managing multiple processes, including inter-process communication and task coordination, which becomes non-negligible when the overall workload is small.

This phenomenon is common in parallel computing and highlights the trade-off between parallel

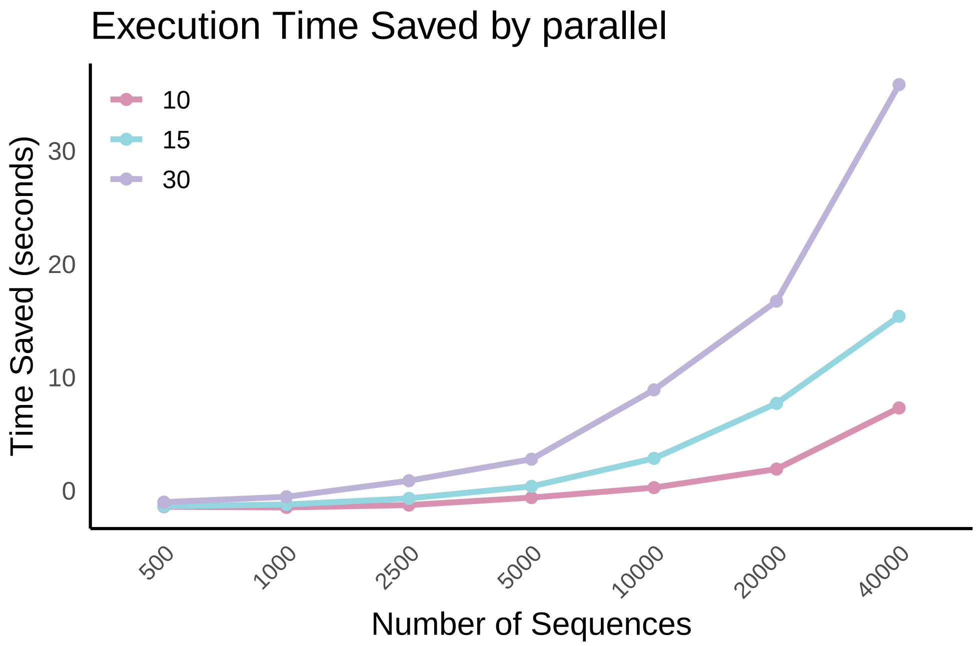

Figure 5: Execution time saved per iteration using the parallel implementation, across various numbers of sequences and sequence lengths.

n	500			1000			2500			5000		
L	10	15	30	10	15	30	10	15	30	10	15	30
Serial	0.68	0.82	1.17	1.16	1.47	2.34	2.69	3.68	5.93	5.61	6.98	11.06
Parallel	2.12	2.21	2.20	2.66	2.71	2.90	3.98	4.38	5.07	6.24	6.62	8.30
Time Saved	-1.44	-1.39	-1.03	-1.50	-1.24	-0.56	-1.29	-0.70	0.86	-0.63	0.36	2.76

n	10000			20000			40000		
L	10	15	30	10	15	30	10	15	30
Serial	11.33	15.05	23.60	22.33	29.79	42.48	44.55	59.67	85.26
Parallel	11.08	12.22	14.72	20.44	22.10	25.77	37.26	44.29	49.43
Time Saved	0.25	2.83	8.88	1.89	7.69	16.71	7.29	15.38	35.83

Table 5: Execution time and time saved (Serial - Parallel) across sequence counts and lengths

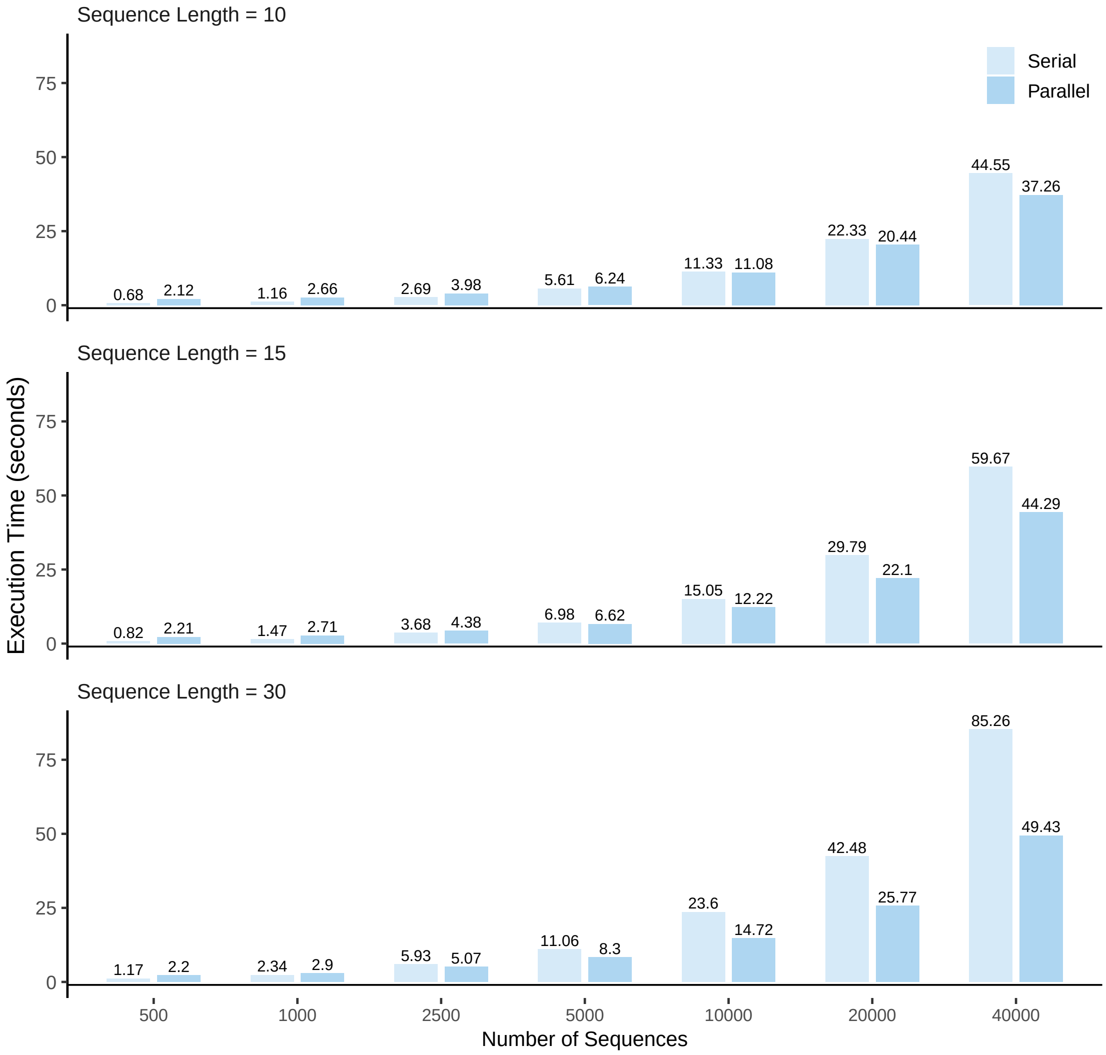

Figure 6: Comparison of average execution time per iteration between serial and parallel implementations different numbers of sequences and sequence lengths.

efficiency and task granularity. In our case, the parallel approach proves most effective for medium to large datasets, where the computational workload per core is sufficient to offset the overhead associated with parallelization. For small-scale datasets, however, the serial implementation remains more efficient and is therefore recommended in such scenarios.

12. Sensitivity analysis on prior settings for the central bulging effect

To examine the robustness of our model with respect to the choice of prior, we conducted a sensitivity analysis on the Gamma prior placed on the Poisson rate parameter λ that governs the distribution of inter-motif gap lengths. In our model, the gap between each pair of adjacent motif positions is modeled as a Poisson random variable with rate λ, where the rate λ follows a Gamma prior $\lambda \sim \text{Gamma}(\beta, \nu)$.

The default prior setting in our model uses $\beta = 1$ and $\nu = 1$, which corresponds to an Exponential distribution with mean 1 and variance 1. To assess the sensitivity of the inferred gap distributions to this prior, we considered four additional configurations of the Gamma prior by altering the values of shape parameter β and rate parameter ν. These settings were chosen to vary both the prior mean and variance in a controlled manner.

The second setting uses $\beta = 2$ and $\nu = 1$, resulting in a prior with mean 2 and variance 2. This setting expresses a prior belief that favors longer average gap lengths. The third setting uses $\beta = 1$ and $\nu = 2$, producing a prior with mean 0.5 and variance 0.25, which encourages shorter gap lengths with higher certainty. The fourth setting adopts $\beta = 0.5$ and $\nu = 0.5$, yielding a prior with mean 1 and variance 2, thus introducing greater uncertainty around the expected gap length. The fifth setting uses $\beta = 2$ and $\nu = 2$, which results in a prior with mean 1 and variance 0.5, representing a more concentrated belief around moderate gap lengths.

For each prior configuration, we applied our model to the real HLA class I peptide datasets corresponding to HLA-A*02:01, HLA-A*24:02, and HLA-A*11:01. In each case, we used a motif length of 8, which was previously determined to be optimal. For each peptide, we estimated the positions of the 8 binding sites and computed the corresponding 9 gap values: 7 internal gaps between adjacent binding positions, as well as 2 flanking gaps between the sequence termini and the nearest binding positions. We then averaged each of these gap values across all peptides of the same length, for each sequence length from 8 to 14.

The gap distributions inferred under each prior setting are visualized in Figures 7, 8, 9, 10, and 11. Each figure includes plots for all three alleles across peptide lengths ranging from 8 to 14. The x-axis indicates the index of each gap segment (from the start of the sequence to the first binding position, through internal motif positions, to the gap between the last binding position and the end of the sequence). The y-axis shows the average gap length for each segment.

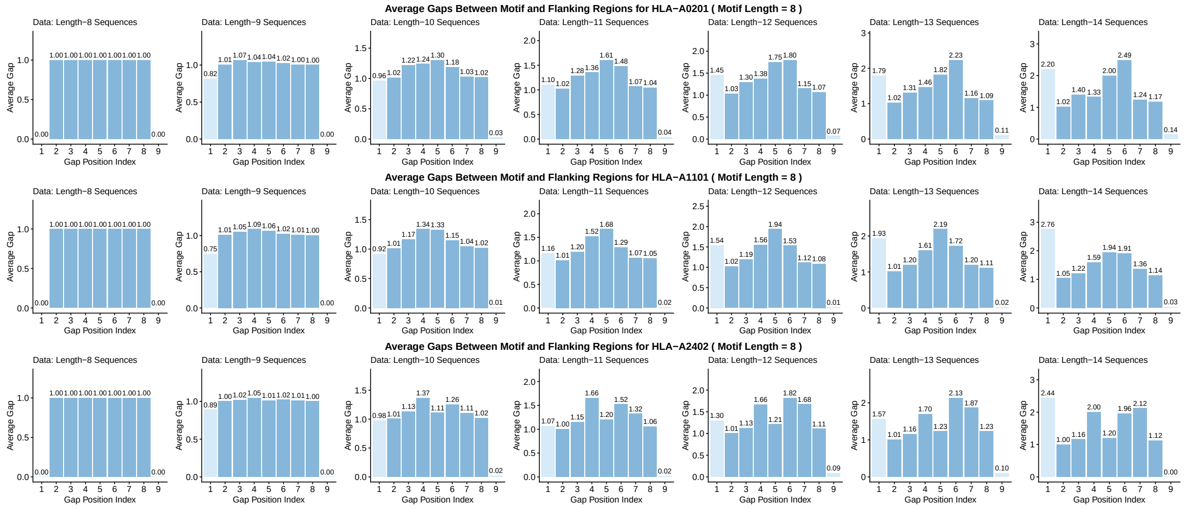

Figure 7: Gap distributions under prior $\lambda \sim \text{Gamma}(1, 1)$ (mean = 1, variance = 1).

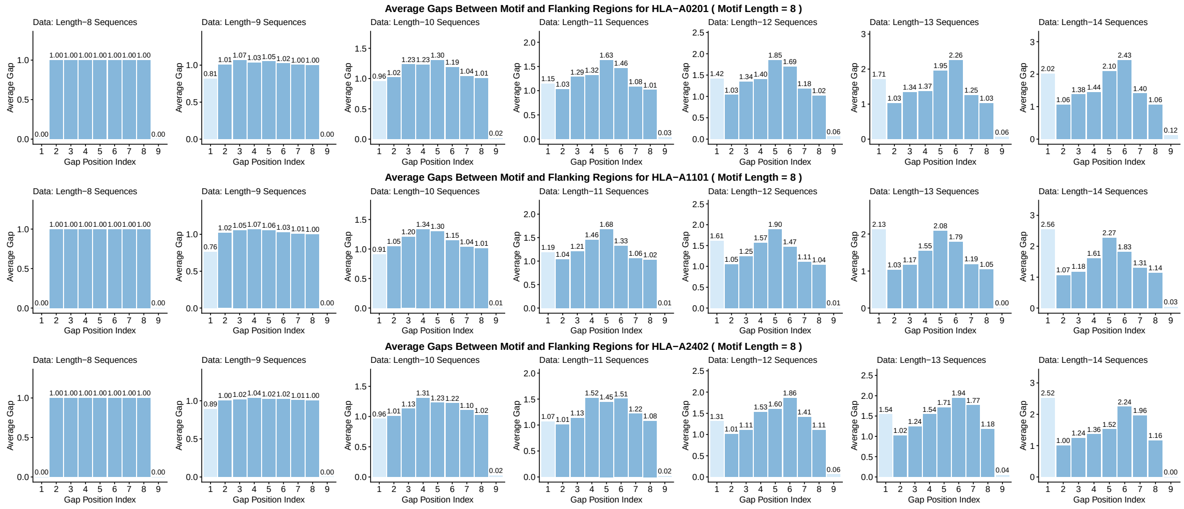

Figure 8: Gap distributions under prior $\lambda \sim \text{Gamma}(2, 1)$ (mean = 2, variance = 2).

Across all five prior settings, we observe a consistent pattern of central expansion in the inferred gap structures. As peptide length increases, the internal gaps located near the center of the motif tend to increase. This suggests a structural preference in which the central region of the

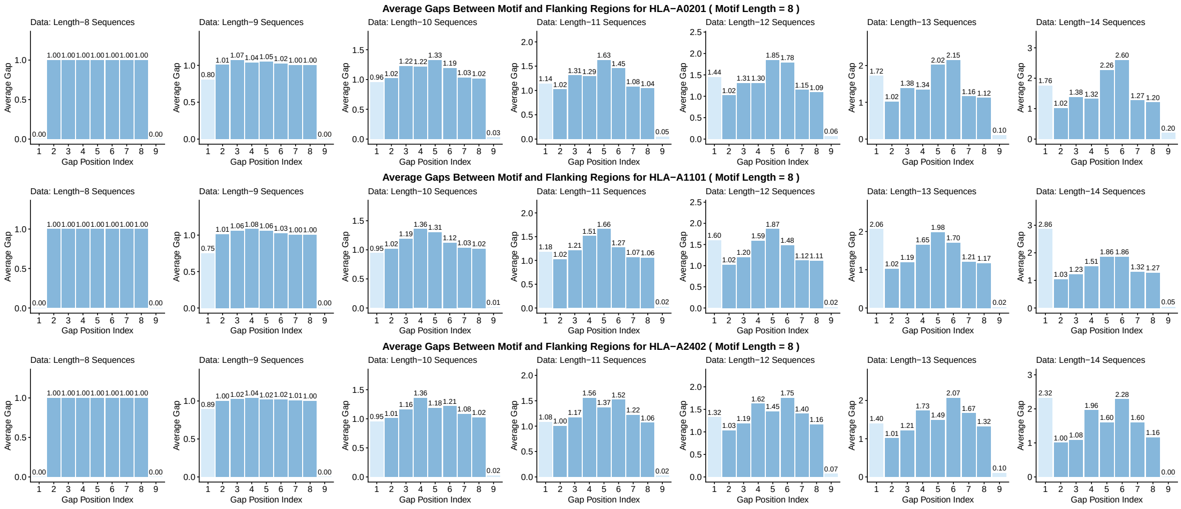

Figure 9: Gap distributions under prior $\lambda \sim \text{Gamma}(1, 2)$ (mean = 0.5, variance = 0.25).

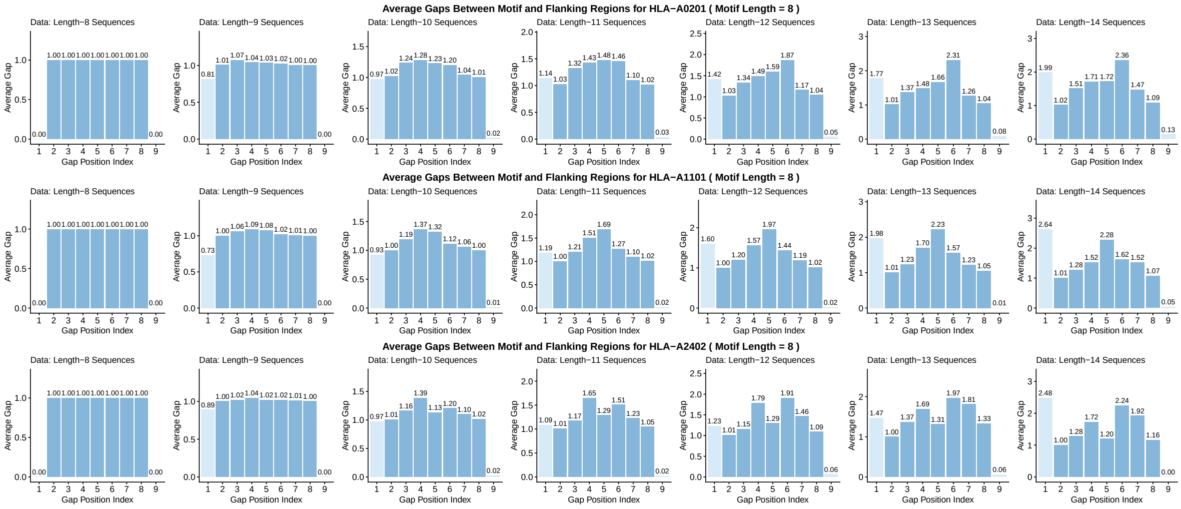

Figure 10: Gap distributions under prior $\lambda \sim \text{Gamma}(0.5, 0.5)$ (mean = 1, variance = 2).

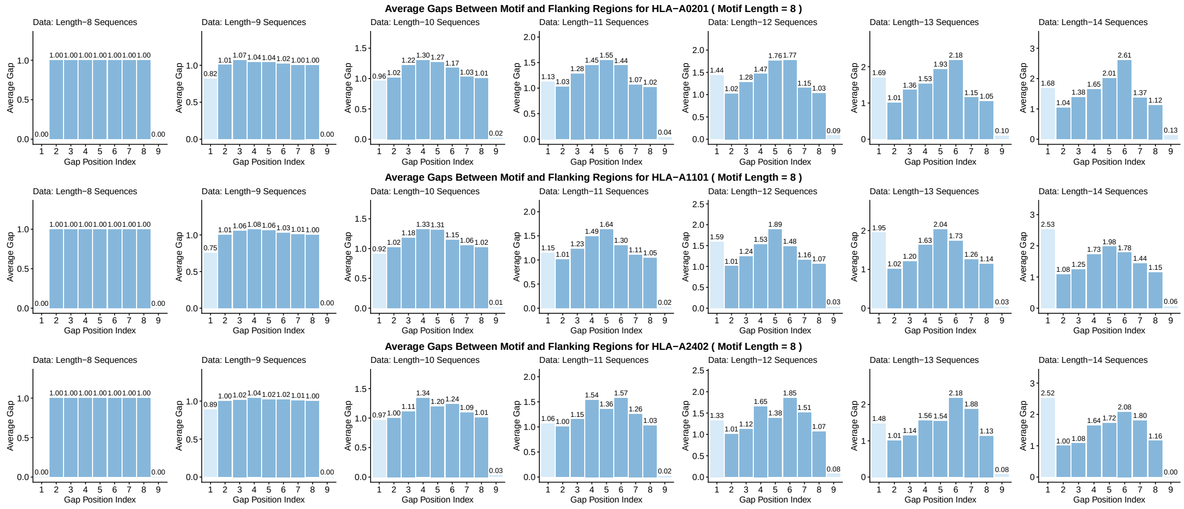

Figure 11: Gap distributions under prior $\lambda \sim \text{Gamma}(2, 2)$ (mean = 1, variance = 0.5).

peptide exhibits greater spatial flexibility as sequence length grows. The central bulging effect is consistently recovered regardless of the choice of Gamma prior, indicating that it is a robust and data-driven feature rather than a consequence of specific prior assumptions.

13. Evaluation on MHC class II binding data

To explore the applicability of our method to MHC class II data, we performed an additional experiment on peptide binding motifs for three of the most prevalent MHC-II alleles in the Chinese population: HLA-DRB1*09:01, HLA-DRB1*15:01, and HLA-DRB1*07:01. Compared to MHC class I, MHC-II molecules bind longer peptides and exhibit open-ended binding grooves, leading to increased variability in the binding core and flanking regions.

We followed the same partially supervised setting as in the main experiments and evaluated prediction accuracy across a range of candidate motif lengths. As shown in Table 6, the best accuracy was achieved at motif length 9 for DRB1*07:01 and DRB1*09:01, and length 10 for DRB1*15:01.

Table 6: Prediction accuracy of peptide binding for different DRB1 alleles across various motif lengths.

**DRB1 Allele**	**Motif Length**											
	**2**	**3**	**4**	**5**	**6**	**7**	**8**	**9**	**10**	**11**	**12**	**13**
DRB1*07:01	0.56	0.591	0.62	0.614	0.642	0.666	0.693	**0.741**	0.712	0.686	0.708	0.711
DRB1*09:01	0.562	0.6	0.598	0.629	0.637	0.678	0.661	**0.729**	0.701	0.697	0.695	0.668
DRB1*15:01	0.558	0.583	0.623	0.663	0.66	0.698	0.7	0.725	**0.794**	0.742	0.721	0.715

Figure 12 compares the sequence logos of the inferred motifs (top row) with reference motifs (bottom row) derived from aligned 9-mer binding cores, as provided by the MHC Motif Atlas. The inferred motifs largely recover the major conserved positions observed in the reference motifs, indicating that our method can extract meaningful binding preferences even in the more challenging MHC-II setting.

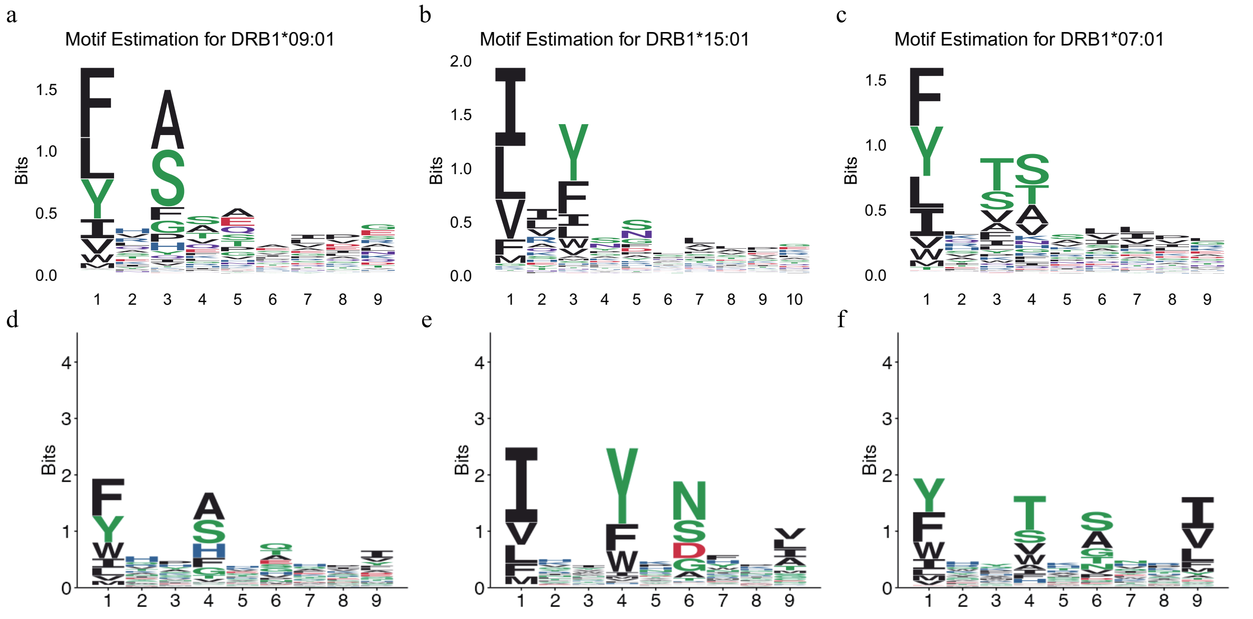

Figure 12: Comparison between inferred and reference motifs for three common MHC-II alleles. The top row (ac) shows the motifs inferred by our model at the optimal motif length for each allele. The bottom row (df) shows the corresponding reference sequence logos derived from aligned 9-mer binding cores, as provided by the MHC Motif Atlas.

Some discrepancies are observed, particularly at less conserved positions. These may arise from two factors. First, MHC-II binding involves longer peptides with flexible, open-ended binding grooves, which increases the uncertainty in motif localization. Second, the reference motifs are constructed under the assumption that the binding core is a contiguous 9-mer aligned across peptides, whereas the actual binding pattern may involve gapped or misaligned positions. Thus, the reference logos serve only as an approximate standard rather than definitive ground truth.

Compared to MHC-I, the prediction accuracy on MHC-II alleles is relatively lower. This trend is consistent with existing MHC-II prediction methods, which generally exhibit reduced performance due to the increased complexity of MHC-II binding. In future work, we plan to refine the model by incorporating prior knowledge about binding core localization, such as positional priors or boundary

regularization, to better accommodate the positional uncertainty and flexible motif boundaries characteristic of MHC-II peptides.

14. Evaluation on JASPAR dataset: GATA1::TAL1

To assess the broader applicability of our model beyond MHC-related motifs, we tested it on the well-characterized transcription factor (TF) binding site dataset GATA1::TAL1 from JASPAR (Matrix ID: MA0140.2). This composite DNA motif consists of two distinct submotifs, GATA1 and TAL1, separated by a low-information gap region, making it a suitable benchmark for evaluating whether our model can identify multiple motif components while excluding intermediate non-informative regions.

We followed the same partially supervised setting as in the main experiments and evaluated prediction accuracy across a range of candidate motif lengths. As shown in Table 7, the prediction accuracy increases with motif length and peaks at lengths 15 and 16. At these lengths, we extracted the inferred motif sequences and compared them to the ground-truth motif from JASPAR (Figure 13). The learned sequence logos closely resemble the expected binding pattern, successfully capturing both the GATA1 and TAL1 submotifs. Moreover, the model correctly omits the low-information region between the submotifs, demonstrating its ability to exclude non-informative gaps.

Table 7: Prediction accuracy of transcription factor binding for GATA1::TAL1 across various motif lengths.

	**Motif Length**									
	2	3	4	5	6	7	8	9	10	11
GATA1_TAL1	0.502	0.5242	0.5222	0.5716	0.6925	0.6825	0.8034	0.7772	0.8185	0.8619
	12	13	14	15	16	17	18			
GATA1_TAL1	0.9002	0.8931	0.9637	**0.9768**	**0.9829**	0.9042	0.9123			

However, we also observe that the inferred motifs contain some additional positions with moderate information content that are not present in the benchmark motif. This may reflect differences in the structural characteristics of DNA sequence data, such as longer motif lengths and fewer residue types (A/C/G/T) compared to peptide sequences. These differences may influence the model's behavior in motif boundary detection. While the current results demonstrate good adaptability of our method to DNA data, further refinements, such as incorporating more tailored priors

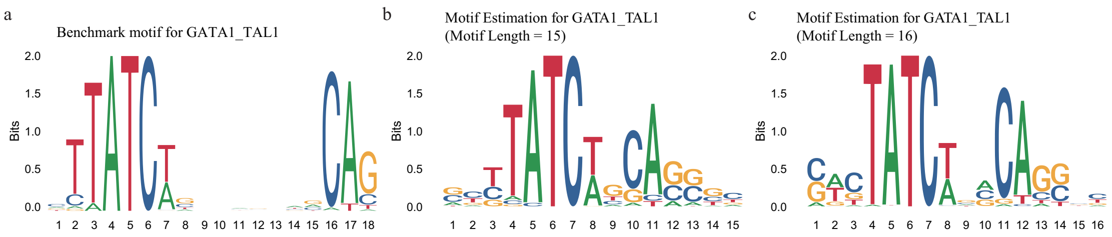

Figure 13: Comparison of the true GATA1::TAL1 motif (a) with the motifs learned by our model at motif lengths 15 (b) and 16 (c). The true motif contains two submotifs separated by a low-information gap. Our model correctly identifies this gap as non-informative and excludes it from the inferred motif region, resulting in a single contiguous logo that accurately captures both submotifs.

or introducing structure-aware constraints, may help improve motif specificity for such sequence types.
